# Asymmetrical Diketopyrrolopyrrole Derivatives with Improved Solubility and Balanced Charge Transport Properties

**DOI:** 10.3390/molecules29122805

**Published:** 2024-06-12

**Authors:** Antonio Carella, Alessandro Landi, Matteo Bonomo, Fabio Chiarella, Roberto Centore, Andrea Peluso, Stefano Nejrotti, Mario Barra

**Affiliations:** 1 Dipartimento di Scienze Chimiche, Università degli Studi di Napoli ‘Federico II’, Complesso Universitario Monte Sant’Angelo, Via Cintia 21, 80126 Napoli, Italy; roberto.centore@unina.it; 2 Department of Chemistry and Biology, University of Salerno, Via Giovanni Paolo II, 84084 Fisciano, Italy; alelandi1@unisa.it (A.L.); apeluso@unisa.it (A.P.); 3 Department of Chemistry, University of Torino, Via Pietro Giuria 7, 10125 Torino, Italy; matteo.bonomo@unito.it (M.B.); stefano.nejrotti@unito.it (S.N.); 4 Nanomaterials for Industry and Sustainability (NIS) Interdepartmental Centre, Via G. Quarello 15A, 10135 Torino, Italy; 5 CNR-Institute for Superconductors, Innovative Materials, and Devices, Dipartimento di Fisica “Ettore Pancini”, P. le Tecchio, 80, 80125 Napoli, Italy; fabio.chiarella@spin.cnr.it (F.C.); mario.barra@spin.cnr.it (M.B.)

**Keywords:** diketopyrrolopyrrole, organic semiconductors, DFT, organic transistors, electrochemistry

## Abstract

The diketopyrrolopyrrole (DPP) unit represents one of the building blocks more widely employed in the field of organic electronics; in most of the reported DPP-based small molecules, this unit represents the electron acceptor core symmetrically coupled to donor moieties, and the solubility is guaranteed by functionalizing lactamic nitrogens with long and branched alkyl tails. In this paper, we explored the possibility of modulating the solubility by realizing asymmetric DPP derivatives, where the molecular structure is extended in just one direction. Four novel derivatives have been prepared, characterized by a common dithyenil-DPP fragment and functionalized on one side by a thiophene unit linked to different auxiliary electron acceptor groups. As compared to previously reported symmetric analogs, the novel dyes showed an increased solubility in chloroform and proved to be soluble in THF as well. The novel dyes underwent a thorough optical and electrochemical characterization. Electronic properties were studied at the DFT levels. All the dyes were used as active layers in organic field effect transistors, showing balanced charge transport properties.

## 1. Introduction

Organic electronics has gained a great deal of interest in recent years both from the academic and industrial world [1,2,3,4]. The motivations for the considerable appeal of this technology lie in the specific features of materials that exhibit the electrical and optical properties of metals and semiconductors while keeping the mechanical and processing characteristics of polymers and organic molecules, in particular a high degree of flexibility [5,6], which leads to an increased resistance towards fracture while allowing to finely modulate the performance of the device [7]. This combination of properties opens the way, from one side, to low-cost manufacturing techniques and, from the other, to novel possible applications not affordable for silicon-based electronics [8,9,10,11,12,13]. In addition, a further strength of this area of research is the possibility of fine-tuning electrical, optical, and other auxiliary properties of organic semiconductors by a proper molecular design [14,15,16,17,18,19]. The last years have witnessed the development of a huge number of new materials potentially promising for applications in several fields of organic electronics, thanks to either experimental study based on empirical intuition [20] or through theoretical design [21,22,23]. In the development of novel organic semiconductors, historical pigments, once mainly investigated for their optical and dyeing properties, have also proved to be useful building blocks for the construction of novel semiconductors, combining good charge transport and processability [24,25]. In this regard, 3,6-diaryl-2,5-dihydro-pyrrolo[3,4-c]pyrrole-1,4-dione, commonly referred to as DPP, represents one of the molecular fragments more investigated in the synthesis of novel organic semiconductors [26,27,28,29]. DPP is a strong electron-deficient unit that has been typically combined with electron donor groups to obtain polymers or small molecules, which demonstrated appealing properties in the field of organic photovoltaics [30,31,32,33,34,35] and organic transistors, as p-type [36,37], n-type [38,39,40], or ambipolar semiconductor [41,42,43]. 

Considering DPP-based small molecules, the DPP unit is typically linked to both sides of donor moieties in a symmetric fashion. The classic strategy to obtain the desired solubility relies on the functionalization of lactam nitrogens of the heterocycle with long and branched alkyl chains. Following this approach, in the last years, we synthesized a novel class of DPP derivatives based on an electron acceptor DPP core symmetrically coupled to donor moieties (bifuran, F-series, or bithiophene, T-series) and end terminated with several auxiliary acceptor groups [44,45]. All the reported materials show a narrow optical bandgap (as low as 1.29 eV). As far as charge transport properties are concerned, almost all these materials displayed ambipolar properties with a slightly predominant n-type charge transport behavior. For what concerns solubility, Furan derivatives proved to be more soluble, as compared to thiophene analogous, so in the latter case we needed to use a longer solubilizing alkyl chain for the functionalization of the lactam nitrogens of the DPP core (2-octyldodecyl vs. 2-ethylhexyl group). Nevertheless, a solubility lower than 10 mg/mL was obtained for T-series dyes. Solubility is a crucial feature in the field of organic electronics; the possibility of fabricating devices by using a solution-based technique is mandatory to keep one of the organic electronics field promises, i.e., lowering the overall processing cost; moreover, solubility is important also in the field of organic photovoltaics, where the active layer is made up of two different materials that need to adopt a proper morphology, with nano-domain phase separation. The topic has been widely investigated both in terms of finding general solubility parameters in organic semiconductor formulations [46] and investigating the effect of designed structural variations in a molecular engineering approach based mainly on the use of different side chains [47,48].

**Scheme 1:**
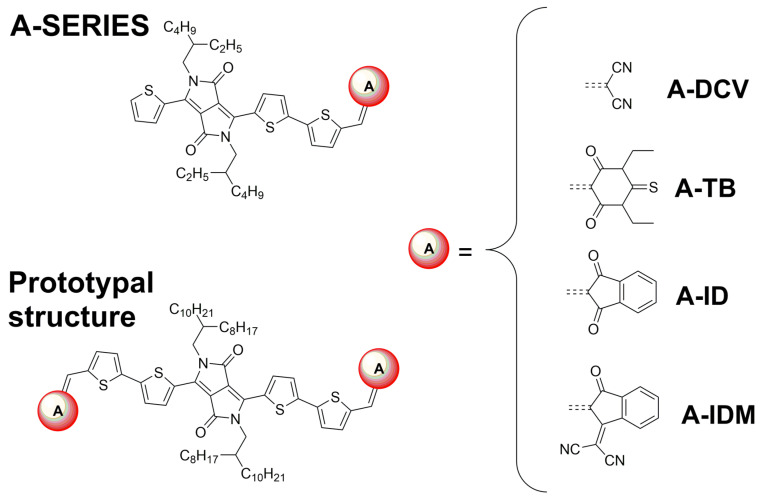
Chemical structures of the A-series dyes studied in this work and the prototypal structure reported in [45].

In this paper, we explore a different approach to improve the solubility of this class of compounds so that they can be solubilized, beyond chlorinated solvents, in more benign solvents as THF. The approach is based on a size reduction and desymmetrization of the prototypal structure, as shown in Scheme 1 (A-series). The common molecular fragment of the new molecules is based on the thienyl DPP unit coupled to the donor thiophene ring; the molecules differ for the end auxiliary acceptor group. The decrease in molecular weight has an obvious effect on reducing the melting point of the molecules (hence increasing the solubility); the lower symmetry has a positive effect on solubility as well, as previously reported [49]. Indeed, the desymmetrization strategy already proved to afford improved solubility in a class of organic semiconductor derivatives applied in organic photovoltaics [50]. This molecular design made it possible to obtain soluble derivatives by using the short 2-ethylhexyl tail (that in the case of the previously reported dyes was sufficient to achieve a good solubility only for furan derivatives), helping the overall synthetic procedure and reducing the percentage of the inactive, insulating part in the final molecular structure. The new dyes were thoroughly characterized in terms of their structural, optical, and thermal properties. Frontier molecular orbital energies were estimated by electrochemical analyses (i.e., cyclic voltammetry). The electronic properties were investigated by a computational analysis performed at the DFT level. The differences in both experimental and computed properties between the new dyes and the analogous symmetric ones are highlighted throughout the papers. The compounds were finally processed as thin films and used as active layers in organic field-effect transistors (OFETs) to investigate their charge transport properties.

## 2. Results and Discussion

### 2.1. Synthesis, Structural Properties, and Solubility

**Scheme 2:**
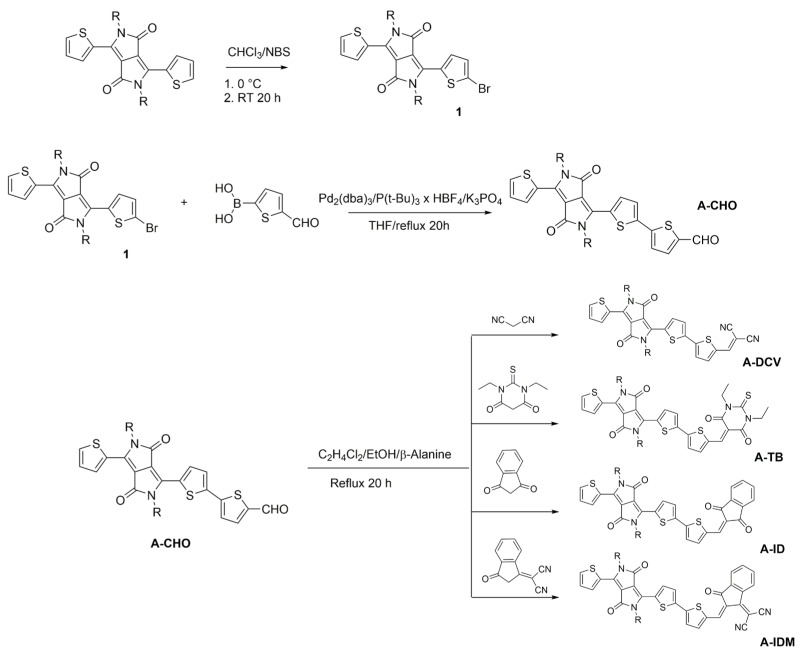
Synthetic strategy for the synthesis of the designed dyes.

The dyes investigated here were synthesized by means of a multi-step synthetic strategy. The synthesis of the DPP core and its *N*-alkylation with 2-ethylhexyl chain was accomplished following reported procedures [45]. Monobromination of the alkylated DPP core was realized by reacting it with *N*-Bromosuccinimide (NBS) in chloroform solution, affording compound **1**. DPP derivative **1** was then reacted with a boronic derivative of 2-formylthiophene in a Suzuki coupling reaction, performed in THF solution, and catalyzed by Pd_2_(dba)_3_. The obtained mono-formyl derivative **A-CHO** was further purified by liquid column chromatography and then used in a Knoevenagel condensation with four different electron acceptor moieties to afford the designed dyes. The dyes were finally purified in hot acetonitrile. The reaction scheme is summarized in Scheme 2.

The identity of the dyes was confirmed by NMR analysis. NMR spectra (shown in the Supporting Information) shared a common signal pattern due to the three thiophene rings linked to the DPP core and a specific resonance associated with the methine peak that shows a clear dependence on the strength of the end acceptor group: the higher the strength, the bigger the d value of this characterizing peak, as a consequence of a stronger de-shielding effect. The strength of the electron-acceptor groups was evaluated as follows: **DCV** < **ID** < **TB** < **IDM**. Moreover, a thermal characterization was carried out on the four dyes: DSC analysis (shown in Figures S25–S28) allowed the determination of the melting temperatures ranging from 155 °C (**A-DCV**) to 228 °C (**A-IDM**), as reported in Table S1. Compared to the symmetrical derivatives described in reference [45], the dyes showed a melting temperature lower than 50 °C (see Figures S25–S28); this decrease is related to the lower molecular weight but could also be the consequence of the less symmetric structure. In previous works, it has been in fact shown that symmetric molecules show higher melting temperature as compared to similar asymmetric ones as a consequence of higher melting enthalpy (shape-cohesion effects) and a residual entropy in the solid state that lower the gain in entropy upon melting [49]. Moreover, all the dyes show a polymorphic behavior. By means of TGA analysis, it was possible to determine the decomposition temperatures T_d_ (defined as the temperature corresponding to 5% weight loss in a TGA scan run in air) of the dyes. A fairly good thermal stability in air, with T_d_ higher than 330 °C, was observed. Thermogravimetric graphs are shown in Figures S29–S32.

It was not possible, unfortunately, to grow single crystals of the dyes suitable for the resolution of the structure through XRD analysis; however, XRD spectra were recorded on drop-casted films of the dyes (see Figure S33). Except for **A-DCV**, whose drop-casted film is prevalently amorphous, the other dyes show several diffraction peaks. In analogy with that proposed with symmetric derivatives, a lamellar organization could be hypothesized with the width of lamella corresponding to the distance associated with the most significant diffraction peaks, found at low 2θ value, corresponding to distances in the range 13–15 Å; these distances are lower than the ones associated with the previously reported symmetric derivatives [45], coherently with the shorter alkyl tails used in the present study.

Solubility was quantitatively measured in chloroform by following the procedure described in the experimental part. As expected, **A-DCV** was the most soluble dye (57 mg/mL), but the other three dyes also showed fairly good solubility, with values as high as 40, 41, and 24 mg/mL, respectively, for **A-TB**, **A-ID**, and **A-IDM**. In contrast, the symmetric analogs of the latter, described in ref. [45], were characterized by a solubility lower than 10 mg/mL. The new compounds present good solubility in other chlorinated solvents, such as dichloromethane and 1,1-2,2-tetrachloroethane, and in more benign solvents, such as THF, as well.

### 2.2. Optical Characterization

**Figure 1:**
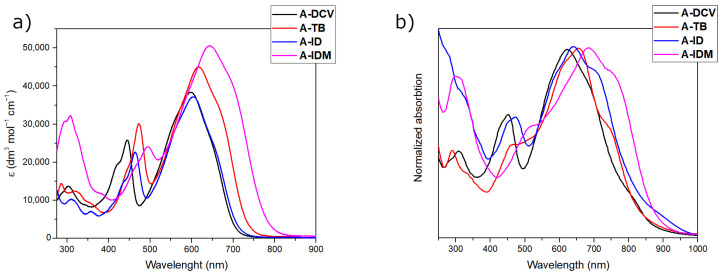
(**a**) Optical absorption spectra of the dyes in chloroform solution (1·10^−5^ M) and (**b**) as thin films spin-coated by chloroform solution and annealed at 100 °C for 1 h.

The dyes were optically characterized in chloroform solution, and the corresponding properties are reported in Table 1. Two main optical features are evident in the UV-Vis spectra of all the dyes, with one in the blue region of each spectrum and the second at longer wavelengths, centered at λ approaching or exceeding 600 nm (see Figure 1a). As already observed in previous papers [44,45,51], absorption maximum positions move to longer wavelengths as the strength of the end acceptor group of the dyes increases. The increase in the electron-acceptor strength improves in fact the π-conjugation length, hence reducing HOMO-LUMO distance and providing an absorption at higher wavelengths. Absorption maximum wavelength values range from 598 nm (**A-DCV**) to 644 nm (**A-IDM**), with **A-TB** and **A-ID** showing intermediate positions at 616 and 604 nm, respectively. Quantitatively, the dyes are good absorbers: molar extinction coefficients (at the longer absorption wavelength) range from 3.72∙10^4^ (**A-DCV**) to 4.63∙10^4^ (**A-IDM**) M^−1^cm^−1^. The obtained values are consistent with the values associated with the symmetric analogous derivatives reported in ref. [45], with the molar extinction coefficient values of the dyes discussed here that are roughly halved. The dyes were also investigated in THF solution: the optical spectra appear similar to those recorded in chloroform with a slight blue shift of the absorption maxima positions (Table 1). As described in the following paragraph, all the dyes show a dipole moment increase upon photoexcitation. Chloroform is slightly more polar than THF and, hence, could better stabilize the polar excited states, leading to higher wavelengths (lower energies) optical transitions.

Optical characterizations were also performed on thin films of the dyes spin-coated from chloroform solutions, as shown in Figure 1b. A common red shift of the absorption at the longer wavelengths is observed. These peaks are characterized by a vibronic structure that emerges more clearly after thermal annealing at 100 °C for 1 h (the difference between optical spectra before and after thermal annealing is shown in Figure S35). By using the Tauc Plot methodology, [52] it is possible to evaluate the optical bandgap of the dyes (see Figures S36–S39). A low bandgap value, lower than or close to 1.5 eV, was found for **A-IDM**, **A-ID**, and **A-TB**, while **A-DCV** experienced a bandgap value of 1.63 eV.

### 2.3. DFT Analysis

**Figure 2:**
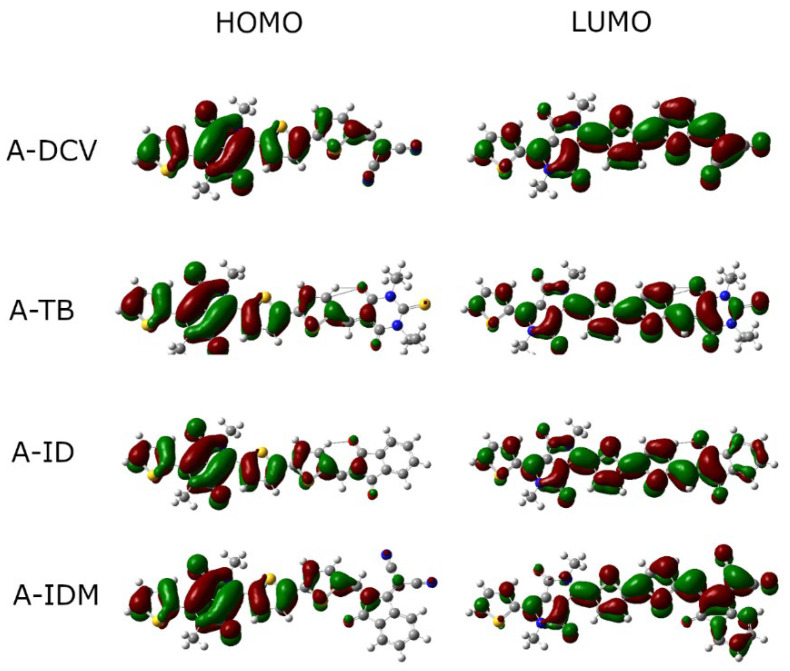
Computed isodensity surfaces of HOMO (left panel) and LUMO (right panel) of the dyes.

To obtain more information about the electronic structure of the synthesized dyes, we performed a computational analysis at the PBE0/6-31+g** level, including solvent effects (chloroform) through the polarizable continuum model. As commonly conducted in these kinds of studies, the side alkyl chains have been modeled as a methyl group [53]. All the molecules have been optimized in their minimal energy structures, where they show a planar conformation. The predicted absorption spectra are reported in the Supporting Information (Figures S40–S43). Wavelengths, energy, and oscillator strengths (larger than 0.1) between ground state orbitals to the excited state are reported in Table S2. The theoretical spectrum in the fifty-excitation range explored by TD-DFT is made up of three/five principal bands, the highest one basically corresponding to the HOMO -> LUMO transition. A good qualitative accord with experimental behavior is observed even though absorption wavelengths are overestimated by about 90–100 nm. In Table 2, we report information about these main transitions, as well as dipole moments of the ground state and of the first excited state, HOMO and LUMO energies, and the optical bandgap obtained from the TD-DFT vertical transition (E_g_). The latter is in good agreement with the experiment, thus validating the calculation and the choice of the density functional. As compared to the analogous symmetric derivatives [45], an increase in LUMO energy of about 0.15 eV is observed, and this could be the consequence of a reduced number of electron acceptor groups in the dyes presented here; a similar but less important effect is observed on HOMO energy as well. As expected, unlike the symmetric analogs, all the chromophores show a clear dipolar nature, less significant in **A-ID**; a consistent increase in the overall dipole moment values is observed when passing from the ground to the excited state. Upon transition to the excited state, electron density moves toward the auxiliary end acceptor groups. HOMO and LUMO density distributions are shown in Figure 2.

### 2.4. Electrochemical Characterization

**Figure 3:**
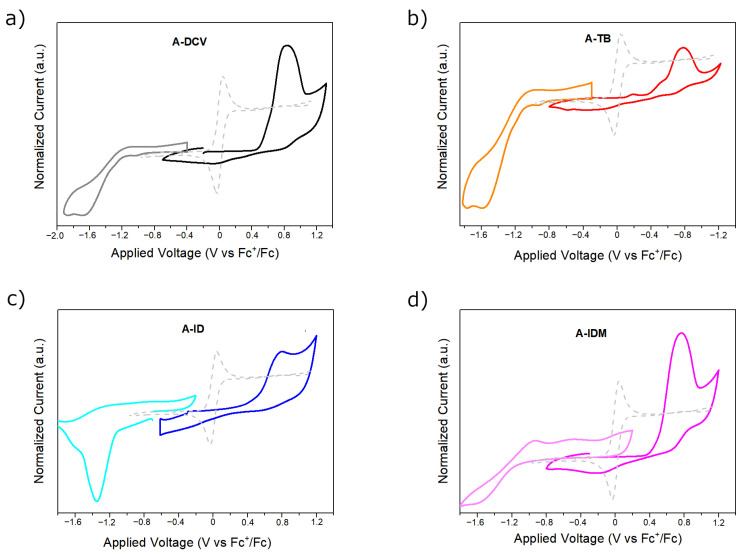
Cyclic voltammetry graphs for: (**a**) **A-DCV**; (**b**) **A-TB**; (**c**) **A-ID**; (**d**) **A-IDM**. In all the curves, the CV scan of ferrocene (dashed light gray line) used as reference is reported.

We performed some cyclovoltammetric (CV) measurements on the dye coated on top of the electrode (see experimental section for further details). The recorded voltammograms are shown in Figure 3. This strategy has been selected in order to emulate the situation the dye will experience once implemented in the final devices. As expected, all CV profiles are characterized by an irreversible peak in the oxidation scan, independently from the nature of the dye, which is characterized by an irreversible oxidation phenomenon. By reporting the potentials relative to the ferrocene/ferrocenium couple, it is possible to estimate HOMO energies by applying the following relation [54] with the measured oxidation potentials:

(1) $$E_{HOMO}\left(eV\right)=-\left(E_{onset}^{ox}+5.1\right)eV$$

The oxidation potentials were measured as the voltage value corresponding to the onset of the oxidation (see Table 3). **A-DCV** presents a single highly irreversible oxidation peak from which a HOMO energy level as high as −5.68 eV could be extracted. **A-TB** (Figure 3b) has a HOMO energy level placed at −5.62, slightly higher than the **A-DCV** counterpart; it also shows an additional (irreversible) peak in the oxidation scan that could be ascribed to the presence of an electronic trap-state (i.e., HOMO-1). **A-ID** and **A-IDM** are characterized by slightly higher HOMO energies of −5.60 eV and −5.58 eV, respectively. From the analysis of the reduction scan of the CV profiles, it was possible to determine the reduction potentials (vs. Fc^+^/Fc) of the dyes, associable with LUMO energies by applying the following [54]:

(2) $$E_{LUMO}\left(eV\right)=-\left(E_{onset}^{red}+5.1\right)eV$$

Also, in this case, reduction potentials were measured by considering the onset of the reduction event. **A-ID** is the only dye characterized by an extremely well-defined (albeit still irreversible) reduction peak (Figure 3c), from which E_LUMO_ = −3.94 eV can be derived. Similar LUMO energies were found for **A-TB** and **A-IDM** (respectively, −3.95 and −3.96 eV), while **A-DCV** features a less stable LUMO (−3.85 eV). The electrochemical bandgaps for all the dyes were calculated by simply subtracting HOMO and LUMO energies, and they are reported in Table 3. Even if larger than optical bandgaps were experimentally found (see Table 1), the trend is perfectly confirmed: **A-IDM** and **A-DCV** are, respectively, the dyes characterized by the lower and the higher bandgaps (1.63 eV and 1.83 eV), while **A-ID** and **A-TB** show intermediate values (1.66 eV for both). The comparison between the reported dyes and the symmetric analogs [45] showed a significant increase in the electrochemically measured LUMO energy (~0.3–0.4 eV), even if a word of caution is needed, considering that in the previous paper, the characterization was performed in solution. This trend, anyway, is coherent with the results emerging from the DFT analysis.

### 2.5. Electrical Characterization

**Figure 4:**
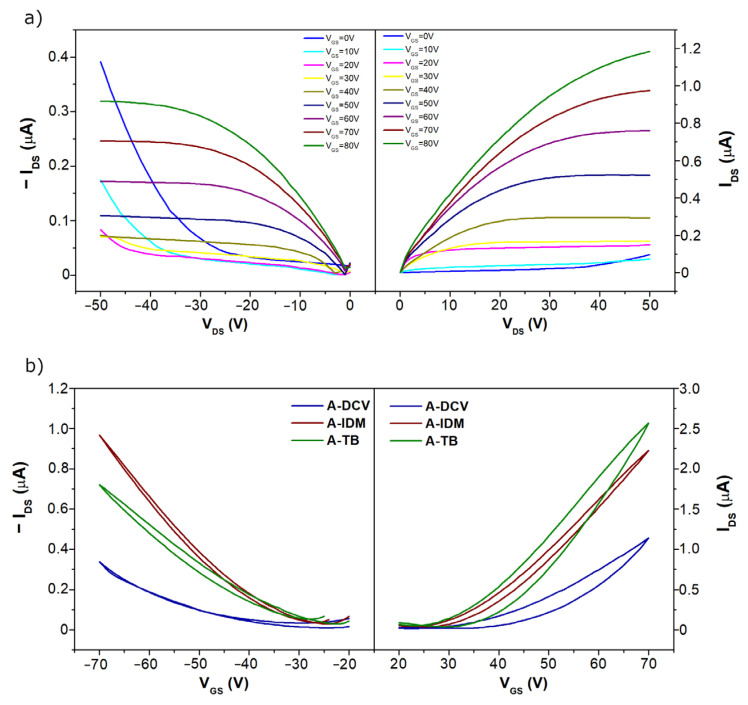
(**a**) Output curves recorded for a **A-IDM** transistor under negative (on the left) or positive (on the right) VGS and VDS voltages. (**b**) Transfer curves recorded in the saturation regime for **A-DCV-**, **A-IDM-**, and **A-TB**-based transistors. On the left and right, the curves are referred to the hole and electron accumulation regions, respectively.

To test their charge transport properties, all the molecules under study were deposed by spin-coating as active layers in OFETs. Except for **A-ID**-based OFETs that did not show any detectable field-effect signal, all the devices analyzed in this study exhibited an ambipolar response, meaning that the current flowing across the channel is increased by applying either positive or negative V_GS_ voltages. This can be clearly inferred from the observation of Figure 4a and Figure S44, showing typical output responses, recorded both in hole (negative V_GS_ and V_DS_) and electron (positive V_GS_ and V_DS_) accumulation regions for transistors based on the synthesized compounds. In these measurements, it is also possible to observe the typical features of ambipolar devices, which emerge at small V_GS_ and large V_DS_ (both considered in absolute values), where the I_DS_ current is not negligible as in unipolar devices but tends to follow a power dependence on V_DS_ being dominated by the minority carriers (electrons and holes for negative and positive V_GS_, respectively) injected from the drain electrode [44].

Figure 4b reports some typical transfer curves measured in the saturation regime for devices based on the various molecules. Here, the I_DS_ current was recorded under negative and positive V_GS_, while V_DS_ was coherently set to −50 V and +50 V, respectively. The transfer curves confirm the ambipolar behavior and show more clearly the occurrence of the hysteretic effects according to which current measured in the forward V_GS_ scan (transistors driven from off to on conditions) is larger than that recorded during the backward scan (transistor from on to off conditions). This type of hysteresis indicates the presence of trapping effects, which tend to immobilize some of the charge carriers during the I_DS_ recording. The transfer curves in Figure 4b also reveal that all the molecules display a prevalent n-type field-effect character with the maximum I_DS_ values under positive V_GS_ (while electrons are accumulated in the channel), being larger in absolute values than those measured under negative V_GS_ (hole accumulation region). Moreover, the compounds **A-IDM** and **A-TB** demonstrated a better electrical response in comparison with **A-DCV**. This general behavior is summarized by mobility and threshold voltage values reported in Table 4.

As shown, in all cases, mobility values (µ_e_) for electrons are higher than those estimated for holes (µ_h_). This is particularly true for **A-IDM** and **A-TB** compounds showing comparable values for µ_e_ (slightly larger than 10^−4^ cm^2^/V·s). If compared with its symmetric counterpart, **A-IDM** is characterized by a significant reduction in µ_e_ by about two orders of magnitude, while µ_h_ decreases by about one order of magnitude [45]. This decrease could be, in part, associated with the increase in LUMO energy, as determined by CV measurements [55]; the asymmetric molecular structure could, in addition, negatively impact the quality of crystal packing, thus reducing charge transport mobility. It is remarkable, anyway, that, in comparison with the corresponding symmetric analog, **A-TB** exhibits very similar electrical performances for the n-type response and even better field-effect response for the hole accumulation region [45] Finally, for **A-DCV**, electron and hole mobility values are much more comparable, being both in the range of 10^−5^ cm^2^/V·s. For all the investigated devices, threshold values in absolute value are close to 20 V. This feature can be mainly ascribed to the presence of residual charge traps, likely associated to the SiO_2_ surface, which must be filled before the charge accumulation phenomenon can take place in the active channel [56].

## 3. Materials and Methods

### 3.1. General Information

All reagents were purchased by Sigma Aldrich or Alfa Aesar and used without any further purification. All the reactions were carried out under a nitrogen atmosphere. 2,5-bis(2-ethylhexyl)-3,6-di(thiophen-2-yl)-2,5-dihydropyrrolo[3,4-c]pyrrole-1,4-dione was prepared following the procedure described in previous work [45]. NMR analysis was employed to confirm the compounds’ identity using Varian Inova 500 (Palo Alto, CA, USA) and Bruker 400 MHz Avance NMR spectrometers (Billerica, MA, USA). Positive Reflectron MALDI spectra were recorded on an AB Sciex TOF/TOF 5800 instrument (Framingham, MA, USA) using 2,5-dihydroxybenzoic acid as the matrix. Thermal stability of the dyes was investigated by means of thermogravimetric analysis (TGA), performed by a TA4000 Perkin-Elmer instrument (Waltham, MA, USA) at a heating rate of 20 °C min^−1^ under air in the temperature range 30–900 °C. The reported decomposition temperature refers to a 5% weight loss. Differential scanning calorimetry (DSC) analysis was performed on a Hitachi NEXTA DSC 200 instrument (Tokyo, Japan) under a nitrogen flow at a heating rate of 10 °C min^−1^. Wide angle X-ray diffraction (WAXD) profiles were obtained with a Malvern Panalytical Empyrean multipurpose diffractometer (Malvern, UK) using Cu Kα radiation (λ = 1.5418 Å) by a continuous scan of the diffraction angle 2θ in the interval 2–40° at a speed of 0.05252 °/s. The diffraction experiments were conducted on thin films of the dye drop-casted on glass substrates (microscope coverslip) by chloroform solution (20 mg/mL). Absorption spectra were recorded on a JASCO V-750 UV-Vis spectrophotometer (Tokyo, Japan) at room temperature with a scanning rate of 200 nm/min, both in solution and as thin films deposed by spin-coating on quartz substrates by means of a Laurell WS-650Mz-23NPP spin processor (Lansdale, PA, USA). FTIR spectra of the dyes were recorded on KBr pellets using a JASCO FTIR 4700 spectrometer. A conventional three-electrode set-up made of a AgNO_3_/Ag reference electrode, a Pt-spiral as counter-electrode, and a custom-made working electrode was used to perform electrochemical measurements; specifically, cyclic voltammetry was performed on the dye’s thin films deposed by drop-casting a solution of each dye (around 1 mg/mL in DCM) on top of a carbon-black electrode and allowing the solvent to evaporate; the deposition step was performed three consecutive times in order to produce a homogeneous film. For each scan (oxidation and reduction), a new working electrode was prepared. Acetonitrile and t-butylammonium-hexafluorophosphate were used as the solvent and supporting electrolyte, respectively. Scan speed was fixed at 50 mV/s, and a positive (negative) direction was imposed for the oxidation (reduction) scan. Due to the poor reversibility of the electrochemical oxidation (e.g., the film is detached from the electrode), only the first cycle of each scan is reported.

### 3.2. Synthesis

#### 3.2.1. Synthesis of 3-(5-Bromothiophen-2-yl)-2,5-bis(2-ethylhexyl)-6-(thiophen-2-yl)-2,5-dihydropyrrolo[3,4-c]pyrrole-1,4-dione (**1**)

A total of 1.84 g (3.50 mmol) of 2,5-bis(2-ethylhexyl)-3,6-di(thiophen-2-yl)-2,5-dihydropyrrolo[3,4-c]pyrrole-1,4-dione was dissolved in 30 mL of chloroform under a nitrogen atmosphere. A total of 520 mg (2.90 mmol) of *N*-bromosuccinimide was added at 0 °C. The system was reacted overnight at room temperature. The day after, the solution was poured in 200 mL of a 0.1 M NaHCO_3_ aqueous solution and extracted with 200 mL of dichloromethane (twice). The organic phase was dried with Na_2_SO_4_, and the solvent was removed by rotary evaporator. The crude product was finally purified by liquid chromatography on silica gel using dichloromethane as an eluent. Yield: 63%. M.p.: 125 °C. 1H-NMR (CD_2_Cl_2_, 400 MHz): δ (ppm) 0.78–0.94 (m, 12H); 1.11–1.43 (m, 16H); 1.88 (s broad, 2H); 3.80–4.06 (m, 4H), 7.25 (d, 1H, *J* = 4.0 Hz); 7.28 (dd, 1H, *J*_1_ = 5.2, *J*_2_ = 4.0 Hz); 7.69 (dd, 1H, *J*_1_ = 4.8 Hz, *J*_2_ = 1.2 Hz); 8.61 (d, 2H, *J* = 4.0 Hz); 8.87 (dd, *J*_1_ = 4.0 Hz; *J*_2_ = 1.2 Hz). 13C-NMR (CD_2_Cl_2_, 400 MHz): δ (ppm) 10.2, 13.8, 23.0, 23.5, 28.3, 30.1, 39.1, 45.7, 107.8, 108.2, 118.3, 128.3, 129.9, 130.9, 131.3, 131.5, 134.8, 135.2, 138.6, 140.7, 161.3, 161.5.

MS MALDI-TOF [M + H]^+^ calcd for C_30_H_40_BrN_2_O_2_S_2_ 603.17, found, 603.14.

FTIR (KBr pellets, cm_−1_): ν = 3095, 2960, 2936, 1671, 1565, 1449, 1418, 1233, 1098, 851, 814, 736, 708.

#### 3.2.2. Synthesis of 5-(2,5-Bis(2-ethylhexyl)-3,6-dioxo-4-(thiophen-2-yl)-2,3,5,6-tetrahydropyrrolo[3,4-c]pyrrol-1-yl)thiophene-2-carbaldehyde (**A-CHO**)

In total, 1.0 g (1.6 mmol) of **1** and 0.516 g (3.2 mmol) of (5-formylthiophen-2-yl)boronic acid were dissolved in 30 mL of anhydrous THF in a 100 mL two-neck round flask; the system was kept under a nitrogen atmosphere. Potassium phosphate tribasic aqueous solution (1.025 g in 3.5 mL of distilled water) was then added. The mixture was degassed for 10 min, then 36 mg of Tris(dibenzylideneacetone)dipalladium(0) [Pd_2_(dba)_3_] and 23 mg of co-catalyst tri-tert-butylphosphonium tetrafluoroborate [P(t-Bu)_3_ × HBF_4_] were added. The system was refluxed overnight and then cooled down to room temperature; THF was evaporated by using a rotary evaporator, and the product was collected by extraction with chloroform/brine and then chloroform/water. The organic phase was collected and dried with anhydrous Na_2_SO_4_ and the solvent evaporated at reduced pressure. The crude product was finally purified by liquid chromatography on silica gel using dichloromethane/petroleum ether 1/1 *v*/*v* as an eluent. Yield: 72%.

M.p.: 120 °C.

1H-NMR (CDCl_3_, 400 MHz): δ (ppm) 0.79–0.95 (m, 12H); 1.15–1.44 (m, 16H); 1.87 (broad, 2H); 3.95–4.09 (m, 4H); 7.29 (dd, 1H, *J*_1_ = 5.2, *J*_2_ = 4.0 Hz); 7.39 (d, 1H, *J* = 4.0 Hz); 7.48 (d, 1H, *J* = 4.0 Hz); 7.66 (dd, 1H, *J*_1_ = 4.8 Hz, *J*_2_ = 1.2 Hz); 7.72 (d, 1H, *J* = 4.0 Hz); 8.88 (d, 1H, *J* = 4.0 Hz); 8.94 (dd, 1H, *J*_1_ = 3.2 Hz, *J*_2_ = 1.2 Hz); 9.90 (s, 1H). 13C-NMR (400 MHz, CD_2_Cl_2_): δ (ppm) 10.2, 10.3, 13.8, 23.1, 23.5, 23.6, 28.3, 28.5, 30.2, 30.3, 39.1, 39.3, 45.7, 108.1, 109.1, 125.5, 126.8, 128.1, 128.3, 129.9, 130.7, 131.1, 134.5, 135.4, 136.0, 137.2, 138.7, 140.3, 140.7, 143.0, 144.9, 161.3, 161.5, 182.4.

MS MALDI-TOF [M + H]^+^ calcd for C_35_H_43_N_2_O_3_S_3_ 635.24, found, 635.22.

FTIR (KBr pellets, cm^−1^): ν = 3091, 2958, 2928, 1666, 1560, 1442, 1419, 1404, 1222, 1104, 1053, 735.

#### 3.2.3. Synthesis of 2-((5-(2,5-Bis(2-ethylhexyl)-3,6-dioxo-4-(thiophen-2-yl)-2,3,5,6-tetrahydropyrrolo[3,4-c]pyrrol-1-yl)thiophen-2-yl)methylene)malononitrile (**A-DCV**)

In total, 150 mg (0.24 mmol) of **A-CHO**, 48 mg (0.73 mmol) of malononitrile, and 43 mg (0.48 mmol) of β-alanine were dissolved in a mixture made up of 9 mL of anhydrous 1,2-dichloroethane and 3 mL of ethanol in a 50 mL two-neck round flask; the solution was refluxed overnight under a nitrogen atmosphere. The system was then cooled down to room temperature, and the solvent was evaporated at reduced pressure. The residue was then dissolved in the minimum amount of dichloromethane and poured into 100 mL of methanol. The obtained solid was recovered by filtration and washed with hot acetonitrile. A microcrystalline product was then recovered. Yield: 73%. 

M.p.: 155 °C. 

1H-NMR (CD_2_Cl_2_, 400 MHz): δ (ppm) 0.76–0.93 (m, 12H); 1.13–1.43 (m, 16H); 1.85 (broad, 2H); 3.92–4.05 (m, 4H); 7.28 (dd, 1H, *J*_1_ = 5.2, *J*_2_ = 4.0 Hz); 7.40 (d, 1H, *J* = 4.4 Hz); 7.54 (d, 1H, *J* = 4.4 Hz); 7.69 (d, 1H, *J* = 4.0 Hz); 7.72 (dd, 1H, *J*_1_ = 4.8 Hz, *J*_2_ = 1.2 Hz); 7.80 (s, 1H); 8.6 (d, 1H, *J* = 4.0 Hz); 8.94 (dd, 1H, *J*_1_ = 4.0 Hz, *J*_2_ = 1.2 Hz). 13C-NMR (CD_2_Cl_2_, 400 MHz): δ (ppm) 10.2, 10.3, 13.8, 23.0, 23.5, 23.6, 28.3, 28.5, 30.2, 30.3, 39.1, 39.4, 45.7, 45.8, 108.1, 113.4, 114.1, 125.8, 127.7, 128.4, 129.8, 131.4, 131.7, 134.7, 135.7, 135.9, 138.2, 139.2, 140.0, 141.2, 147.1, 150.0, 161.3, 161.5.

MS MALDI-TOF [M]^+^ calcd for C_38_H_42_N_4_O_2_S_3_ 682.25, found, 682.26.

FTIR (KBr pellets, cm^−1^): ν = 3081, 2959, 2929, 2221, 1667, 1572, 1559, 1427, 1419, 1259, 1066, 800, 731.

#### 3.2.4. Synthesis of 3-(5-((1,3-Diethyl-4,6-dioxo-5-thioxotetrahydropyrimidin-2(1*H*)-ylidene)methyl)thiophen-2-yl)-2,5-bis(2-ethylhexyl)-6-(thiophen-2-yl)-2,5-dihydropyrrolo[3,4-c]pyrrole-1,4-dione (**A-TB**)

In total, 100 mg (0.16 mmol) of **A-CHO**, 100 mg (0.50 mmol) of *N*,*N*-diethylthiobarbituric acid, and 28 mg (0.32 mmol) of β-alanine were dissolved in a mixture made up of 9 mL of anhydrous 1,2-dichloroethane and 3 mL of ethanol in a 50 mL two-neck round flask; the solution was refluxed overnight under a nitrogen atmosphere. The system was then cooled down to room temperature, and the solvent was evaporated at reduced pressure. The residue was then dissolved in the minimum amount of dichloromethane and poured into 100 mL of methanol. The obtained solid was recovered by filtration and washed with hot acetonitrile. A microcrystalline product was then recovered. Yield: 82%.

M.p.: 195 °C.

1H-NMR (CDCl_3_, 500 MHz): δ (ppm) 0.80–0.96 (m, 12H); 1.18–1.46 (m, 22H); 1.89 (broad, 2H); 3.98–4.14 (m, 4H); 4.52–4.70 (m, 4H); 7.29 (t, 1H, *J*_1_ = 4.5 Hz); 7.47 (d, 1H, *J* = 4.0 Hz); 7.62–7.69 (m, 2H); 7.82 (d, 1H, *J* = 4.0 Hz); 8.63 (s, 1H); 8.92 (d, 1H, *J* = 4.0 Hz); 8.96 (d, 1H, *J* = 4.0 Hz). 13C-NMR (CDCl3, 500 MHz): δ (ppm) 10.2, 10.3, 12.1, 12.2, 13.8, 23.1, 23.5, 23.6, 28.4, 28.5, 30.2, 30.3, 39.1, 39.4, 43.1, 43.8, 45.8, 108.1, 109.4, 110.8, 125.8, 127.6, 128.4, 129.9, 131.2, 131.7, 135.6, 136.1, 137.4, 138.5, 140.5, 140.9, 146.8, 148.4, 151.6, 159.8, 160.6, 161.3, 161.5, 178.8.

MS MALDI-TOF [M + Na]^+^ calcd for C_43_H_52_N_4_NaO_4_S_4_ 839.28, found, 839.25.

FTIR (KBr pellets cm^−1^): ν = 2956, 2929, 1663, 1550, 1441, 1391, 1264, 1229, 1104, 1069, 904, 800, 736, 712.

#### 3.2.5. Synthesis of 3-(5-((1,3-Dioxo-1,3-dihydro-2*H*-inden-2-ylidene)methyl)thiophen-2-yl)-2,5-bis(2-ethylhexyl)-6-(thiophen-2-yl)-2,5-dihydropyrrolo[3,4-c]pyrrole-1,4-dione (**A-ID**)

In total, 100 mg (0.16 mmol) of **A-CHO**, 75 mg (0.51 mmol) of 1,3-indandione, and 28 mg (0.32 mmol) of β-alanine were dissolved in a mixture made up of 9 mL of anhydrous 1,2-dichloroethane and 3 mL of ethanol in a 50 mL two-neck round flask; the solution was refluxed overnight under a nitrogen atmosphere. The system was then cooled down to room temperature, and the solvent was evaporated at reduced pressure. The residue was then dissolved in the minimum amount of dichloromethane and poured into 100 mL of methanol. The obtained solid was recovered by filtration and washed with hot acetonitrile. A microcrystalline product was then recovered. Yield: 81%.

M.p.: 212 °C.

1H-NMR (CDCl_3_, 500 MHz): δ (ppm) 0.77–0.96 (m, 12H); 1.18–1.46 (m, 16H); 1.90 (broad, 2H); 3.99–4.13 (m, 4H); 7.29 (dd, 1H, *J*_1_ = 5.0, *J*_2_ = 4.0 Hz); 7.42 (d, 1H, *J* = 4.0 Hz); 7.59 (d, 1H, *J* = 4.5 Hz); 7.66 (dd, 1H, *J*_1_ = 5.0 Hz, *J*_2_ = 1.0 Hz); 7.76–7.82 (m, 2H); 7.91 (d, 1H, *J* = 4.0 Hz); 7.94 (s, 1H); 7.96–8.01 (m, 2H); 8.93–8.95 (m, 2H). 13C-NMR (CDCl_3_, 500 MHz): δ (ppm) 10.5, 10.6, 14.0, 14.1, 23.1, 23.6, 23.7, 28.4, 28.5, 30.2, 30.3, 39.1, 39.3, 46.0, 108.2, 109.2, 122.9, 123.1, 124.6, 125.9, 127.1, 128.5, 129.7, 130.8, 131.0, 135.0, 135.2, 135.8, 136.6, 137.5, 138.9, 140.5, 140.9, 142.0, 143.2, 148.0, 161.5, 161.6, 189.6, 189.9.

MS MALDI-TOF [M + Na]^+^ calcd for C_44_H_46_N_2_NaO_4_S_3_ 785.25, found, 785.21.

FTIR (KBr pellets, cm^−1^): ν = 3068, 2961, 2928, 1674, 1579, 1543, 1432, 1261, 1100, 1020, 800, 732.

#### 3.2.6. Synthesis of (*E*)-2-(2-((5-(2,5-Bis(2-ethylhexyl)-3,6-dioxo-4-(thiophen-2-yl)-2,3,5,6-tetrahydropyrrolo[3,4-c]pyrrol-1-yl)thiophen-2-yl)methylene)-3-oxo-2,3-dihydro-1*H*-inden-1-ylidene)malononitrile (**A-IDM**)

In total, 100 mg (0.16 mmol) of **A-CHO**, 100 mg (0.51 mmol) of 1,1-dicyanomethylene-3-indanone, and 28 mg (0.32 mmol) of β-alanine were dissolved in a mixture made up of 9 mL of anhydrous 1,2-dichloroethane and 3 mL of ethanol in a 50 mL two-neck round flask; the solution was refluxed overnight under a nitrogen atmosphere. The system was then cooled down to room temperature, and the solvent was evaporated at reduced pressure. The residue was then dissolved in the minimum amount of dichloromethane and poured into 100 mL of methanol. The obtained solid was recovered by filtration and washed with hot acetonitrile. A microcrystalline product was then recovered. Yield: 82%. 

M.p.: 227 °C.

1H-NMR (CDCl_3_, 500 MHz): δ (ppm) 0.76–0.96 (m, 12H); 1.16–1.46 (m, 16H); 1.89 (broad, 2H); 3.97–4.11 (m, 4H); 7.28 (t, 1H, *J* = 4.5 Hz); 7.42 (d, 1H, *J* = 4.5 Hz); 7.58 (d, 1H, *J* = 4.5 Hz); 7.66 (d, 1H, *J* = 5.0 Hz); 7.73–7.80 (m, 3H); 7.92–7.97 (m, 1H); 8.63–8.69 (m, 1H); 8.81 (s, 2H); 8.92–8.98 (m, 2H). 13C-NMR (CDCl_3_, 500 MHz): δ (ppm) 10.4, 10.5, 14.0, 23.1, 23.5, 23.6, 28.3, 28.6, 30.2, 30.3, 39.1, 39.3, 46.0, 108.1, 109.4 114.0, 123.1, 123.8, 125.7, 127.7, 128.6, 129.7, 131.3, 131.5, 134.6, 135.3, 136.1, 136.7, 136.9, 137.1, 138.5, 139.9, 140.5, 141.1, 145.7, 150.77, 159.7, 161.2, 161.5, 188.1.

MS MALDI-TOF [M]^+^ calcd for C_47_H_46_N_4_O_3_S_3_ 810.27, found, 810.25.

FTIR (KBr pellets, cm^−1^): ν = 2958, 2929, 2216, 1704, 1668, 1544, 1438, 1388, 1330, 1226, 1132, 1065, 989, 801, 718.

### 3.3. Solubility Measurements

For each dye, 70 mg was placed in 1 mL of chloroform and stirred overnight in a closed vial and in the dark. Each suspension was then filtered through a syringe filter (Whatman^®^ Puradisc 13, Little Chalfont, UK). The obtained solutions were diluted 1/3000 and analyzed at the UV-Vis spectrophotometer. From the measured absorbance and the knowledge of the molar extinction coefficients, it was possible to determine the solubility of the compounds.

### 3.4. DFT Studies

All electronic computations were carried out at the density functional level of theory (DFT) by using the PBE0 functional together with the polarized 6–31+G(d,p) basis set [57]. The level of computations should provide reliable results, according to previous work [45]. Geometry optimizations and computations of normal coordinates and harmonic vibrational frequencies were carried out by using the Gaussian package (G16) [58]. To verify that the stationary points found were real minima on the potential energy surface, the calculation of the vibrational frequencies was carried out, and no imaginary values were found in any case. Time-dependent DFT (TDDFT) was employed for treating all excited states and to obtain the UV-Vis absorption spectra. Solvent (chloroform) effect was included in the computation through the Polarizable Continuum Model (PCM), as implemented in the code Gaussian 16 [59]. The long alkyl chains were replaced by methyl groups in all the electronic computations, as routinely done in DFT studies [60,61].

### 3.5. Electrical Characterization

Bottom-contact bottom-gate organic field-effect transistors (OFETs) based on the studied dyes as active layers were fabricated by depositing thin films of the dyes on multilayer structures consisting of a 500 μm thick highly doped silicon (Si^++^), acting as substrate and gate electrode, a 200 nm thick SiO_2_ dielectric barrier, and 150 nm thick pre-patterned interdigitated gold electrodes working as source and drain contacts [62]. This device architecture provides active channels characterized by large width/length (W/L) ratios (~550), making the estimation of charge carrier mobility values down to 10^−7^ cm^2^/V·s possible. The dye’s films were deposited by spin-coating from chloroform solutions (5 mg/mL) at 1000 rpm for 1 min and then at 2000 rpm for 30 s. Prior dye’s deposition, Si/SiO_2_ substrates, were functionalized by HMDS (hexamethyldisilazane) using a previously established procedure [45] to enhance the hydrophobic character of the dielectric surface, hence reducing the effects of water-related charge-trapping mechanisms on the final transistor response. After the dye’s deposition, the devices were annealed for 1 h at 100 °C under vacuum. Electrical characterization of the transistors was performed in vacuum (pressure ~10^−5^ mbar) using a Janis cryogenic probe station connected to a Keithley 2612A dual-channel source meter. It was possible, in this way, to measure currents between drain and source (I_DS_) and between gate and source (I_GS_) while applying different voltages between drain and source (V_DS_) and gate and source (V_GS_). Transfer (I_DS_ vs. V_GS_ at fixed V_DS_) and output (I_DS_ vs. V_DS_ at fixed V_GS_) curves were recorded considering both the electron- (positive V_GS_ and V_DS_) and hole accumulation (negative V_GS_ and V_DS_) regions. From the transfer curves, in the saturation regime, mobility (µ) and threshold voltage (V_th_) values could be extrapolated by linearly fitting the MOSFET equation:

(3) $$\sqrt{\left|I_{DS}\right|}=\sqrt{\frac{W\cdot \mu \cdot C_{ox}}{2L}}\cdot \left|V_{GS}-V_{th}\right|$$

In this equation, C_ox_ is the dielectric oxide capacitance per unit area, equal to 17.25 nF/cm^2^ in our case.

## 4. Conclusions

Four novel DPP derivatives were reported here. Unlike most DPP-based small molecules reported in the literature, the new dyes have been designed so that the thienyl-DPP unit does not represent the core but rather one end of the conjugated molecular structure. This is based on a common molecular fragment constituted by a dithienyl DPP unit, linked from one side to the donor thiophene bearing four different auxiliary acceptor groups. The asymmetrical structure was designed with the aim of increasing the solubility of the derivatives; indeed, as compared to symmetric analogs previously reported by some of us, the new dyes proved to have an increased solubility in chloroform and other chlorinated solvents. Moreover, the new compounds were also soluble in more benign solvents as THF. A thorough optical characterization was conducted both in solution and as thin films. For what concerns optical properties (in CHCl_3_ and in THF as well), all the dyes have high molar absorption coefficients, and their absorption maximum positions are clearly influenced by the strength of the end auxiliary acceptor group. In the solid phase (as thin films), a bathochromic shift of the absorption is observed for all the dyes, and moderately low bandgaps (as low as 1.5 eV) were measured. An electrochemical characterization was performed on thin films of the prepared dyes. From the measurement of oxidation and reduction potentials, it was possible to determine HOMO and LUMO energies of the dyes. As compared to the symmetric analogs previously reported, an increase in both HOMO and LUMO energies can be observed. Electronic properties were investigated at the DFT level. Unlike the symmetric analogs previously reported, as expected, the dyes showed a more pronounced dipolar behavior; the calculations showed that, upon photoexcitation, electron density moves toward the auxiliary end acceptor group. Charge transport properties were studied by depositing the synthesized molecules as active layers in OFETs. All the dyes, except **A-ID**, showed a detectable field-effect response with a prevalent n-type behavior (µ_e_ ~10^−4^ cm^2^ V^−1^ s^−1^) and a slighter lower hole transport ability (µ_h_ ~20–30% of µ_e_). The electron mobilities found are similar to those measured for Y-series molecules, one of the most promising classes of nonfullerene acceptors recently investigated [63].

## Figures and Tables

**Table 1:** Optical properties of the synthesized dyes in solution and as thin film.

Dyes	λ_max_ (nm) in CHCl_3_	ε (dm^3^·mol^−1^·cm^−1^) in CHCl_3_	λ_max_ (nm) in THF	ε (dm^3^·mol^−1^·cm^−1^) in THF	λ_max_ (nm) as Thin Film ^1^	E_g_(eV) ^2^
**A-DCV**	598/445	3.35 × 10^4^/2.23 × 10^4^	592/439	4.51 × 10^4^/2.87 × 10^4^	667/620/451	1.63
**A-TB**	616/473	4.01 × 10^4^/2.72 × 10^4^	610/468	4.88 × 10^4^/3.12 × 10^4^	732/655/515	1.53
**A-ID**	605/464	4.36 × 10^4^/2.75 × 10^4^	599/457	4.46 × 10^4^/2.66 × 10^4^	702/640/473	1.51
**A-IDM**	644/495	4.63 × 10^4^/2.18 × 10^4^	631/489	4.85 × 10^4^/2.19 × 10^4^	746/685/520	1.48

^1^ Spin-coated from a chloroform solution (5 mg/mL) and annealed 1 h at 100 °C; ^2^ Determined by the Tauc plot methodology [52].

**Table 2:** Computed electronic and optical properties of the synthesized dyes.

Dyes	λ_abs_ (nm)	F	µ_GS_ (D)	µ_ES_ (D)	HOMO (eV)	LUMO (eV)	E_g_ (eV)	E_g_^EXP^(eV) ^1^
**A-DCV**	682	1.27	11.90	13.58	−5.64	−3.45	1.82	1.76
**A-TB**	697	1.57	9.19	11.82	−5.62	−3.46	1.78	1.70
**A-ID**	674	1.60	3.27	5.15	−5.56	−3.34	1.84	1.75
**A-IDM**	744	1.46	9.58	11.67	−5.60	−3.57	1.66	1.60

^1^ Measured by determining the onset of the absorption in chloroform solution.

**Table 3:** Electrochemical potentials vs. Fc^+^/Fc and estimated HOMO and LUMO energies vs. vacuum and electrochemical bandgap values.

Dyes ^1^	E_ox_ (V) ^2^	E_red_(V) ^3^	HOMO (eV) ^4^	LUMO (eV) ^5^	E_g_^ec^ (eV) ^6^
**A-DCV**	0.58	−1.25	−5.68	−3.85	1.83
**A-TB**	0.52	−1.14	−5.62	−3.96	1.66
**A-ID**	0.50	−1.16	−5.60	−3.94	1.66
**A-IDM**	0.48	−1.15	−5.58	−3.95	1.63

^1^ Electrochemical measurements were performed on dye’s thin films deposed on the working electrode; ^2^ calculated as the onset of the first oxidation peak; ^3^ calculated as the onset of the first reduction peak; ^4^ determined by applying Equation (1); ^5^ determined by applying Equation (2); ^6^ the electrochemical bandgap (E_g_^ec^) is calculated as |HOMO-LUMO|.

**Table 4:** Electron (µ_e_) and hole (µ_h_) mobility and corresponding threshold voltage (V_th,e_ and V_th,h_) for the transistors based on the synthesized molecules ^1^.

Dyes	µ_e_^aver^ (cm^2/V^·s)	µ_e_^max^ (cm^2/V^·s)	V_th,e_^aver^ (V)	µ_h_^aver^ (cm^2/V^·s)	µ_h_^max^ (cm^2/V^·s)	V_th,h_^aver^ (V)
**A-DCV**	(6.5 ± 3.7)·10^−5^	1.1·10^−4^	21.3 ± 1.5	(1.8 ± 0.9)·10^−5^	2.8·10^−5^	−22.9 ± 0.3
**A-TB**	(1.7 ± 0.7)·10^−4^	2.6·10^−4^	18.4 ± 1.7	(3.7 ± 1.4)·10^−5^	5.2·10^−5^	−16.5 ± 2.7
**A-IDM**	(1.8 ± 0.5)·10^−4^	2.4·10^−4^	15.1 ± 4.8	(6.6 ± 0.4)·10^−5^	7.1·10^−5^	−21.0 ± 2.4

^1^ The values were estimated from the transfer curves in the saturation regime by linearly fitting the MOSFET equation, as described in the Materials and Methods section; average values were achieved by considering at least four transistors for any molecule, while error bars represent the standard deviations.

## Data Availability

Data are contained within the article or Supplementary Material.

## Supplementary Materials

The following supporting information can be downloaded at: https://www.mdpi.com/article/10.3390/molecules29122805/s1: 1H NMR, 13C NMR, Maldi-TOF MS and FTIR spectra for all the precursors and the final dyes (Figures S1–S24); DSC and TGA graphs for **A-DCV**, **A-TB**, **A-ID**, and **A-IDM** molecules (Figures S25–S32); XRD spectra on drop-casted films of the dyes (Figure S33); Optical spectra of the dyes in THF solution and as thin films deposed by THF solution (Figure S34); Effect of annealing procedure on optical features of the dye’s thin films (Figure S35); Tauc plot for **A-DCV**, **A-TB**, **A-ID**, and **A-IDM** molecules (Figures S36–S39); Computed optical spectra for **A-DCV**, **A-TB**, **A-ID**, and **A-IDM** molecules (Figures S40–S43); Output curves for **A-DCV**-and **A-TB**-based transistors (Figure S44); Thermal properties for **A-DCV**, **A-TB**, **A-ID**, and **A-IDM** molecules (Table S1); Optical properties in THF solution (Table S2); Computed main optical transitions in the synthesized dye. Only transitions with Oscillator strength > 0.1 have been reported (Table S3).
