# Supplementary material for: Asymmetrical Diketopyrrolopyrrole Derivatives with Improved Solubility and Balanced Charge Transport Properties
Source: Molecules. 2024 Jun 12;29(12):2805. doi: 10.3390/molecules29122805 (PMC11207042; doi:10.3390/molecules29122805)
Supplement: Supplementary file 1 [file molecules-29-02805-s001.zip › molecules-3041499-supplementary.pdf]

Article

Asymmetrical Diketopyrrolopyrrole Derivatives with Improved Solubility and Balanced Charge Transport Properties

Antonio Carella [1,*], Alessandro Landi [2], Matteo Bonomo [3,4], Fabio Chiarella [5], Roberto Centore [1], Andrea Peluso [2], Stefano Nejrotti [3,4] and Mario Barra [5]

1 Dipartimento di Scienze Chimiche, Università degli Studi di Napoli 'Federico II', Complesso Universitario Monte Sant'Angelo, Via Cintia 21, 80126 Napoli, Italy; roberto.centore@unina.it
2 Department of Chemistry and Biology, University of Salerno, Via Giovanni Paolo II, 84084 Fisciano, Italy; alelandi1@unisa.it (A.L.); apeluso@unisa.it (A.P.)
3 Department of Chemistry, University of Torino, Via Pietro Giuria 7, 10125 Torino, Italy; matteo.bonomo@unito.it (M.B.); stefano.nejrotti@unito.it (S.N.)
4 Nanomaterials for Industry and Sustainability (NIS) Interdepartmental Centre, Via G. Quarello 15A, 10135 Torino, Italy
5 CNR-Institute for Superconductors, Innovative Materials, and Devices, Dipartimento di Fisica "Ettore Pancini", P. le Tecchio, 80, 80125 Napoli, Italy; fabio.chiarella@spin.cnr.it (F.C.); mario.barra@spin.cnr.it (M.B.)
* Correspondence: antonio.carella@unina.it

Supplementary Materials

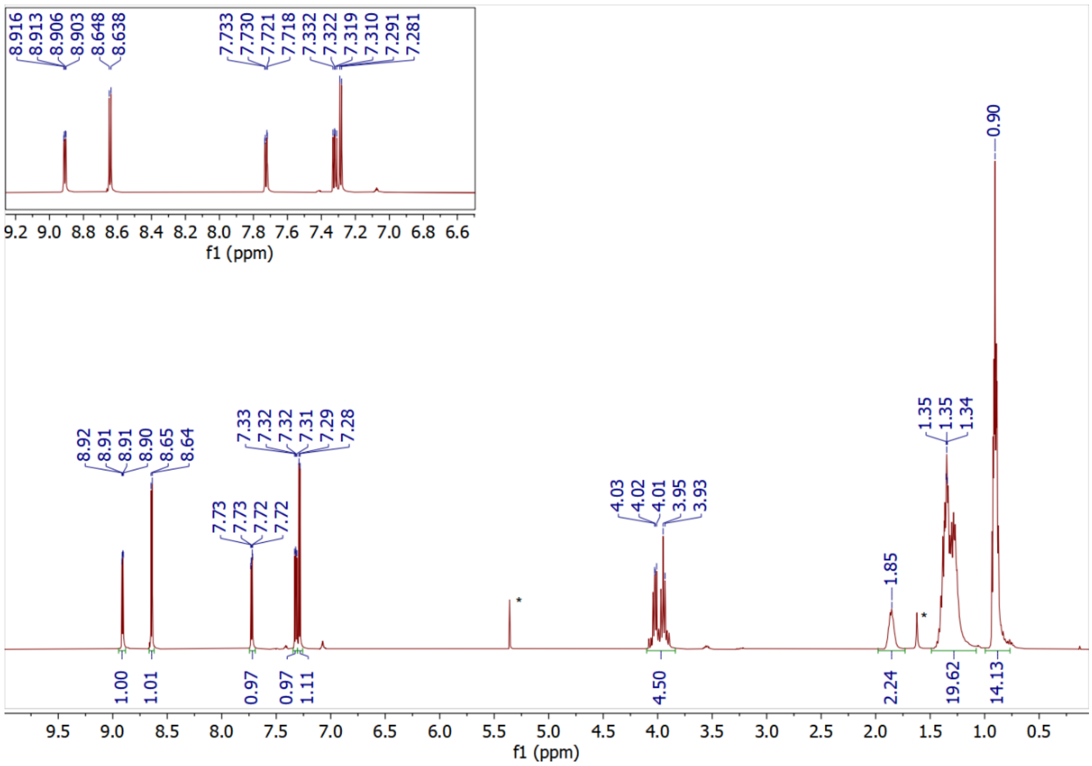

Figure S1. 1H NMR of compound **1** in CD_2Cl_2. Signals relative to solvents are starred.

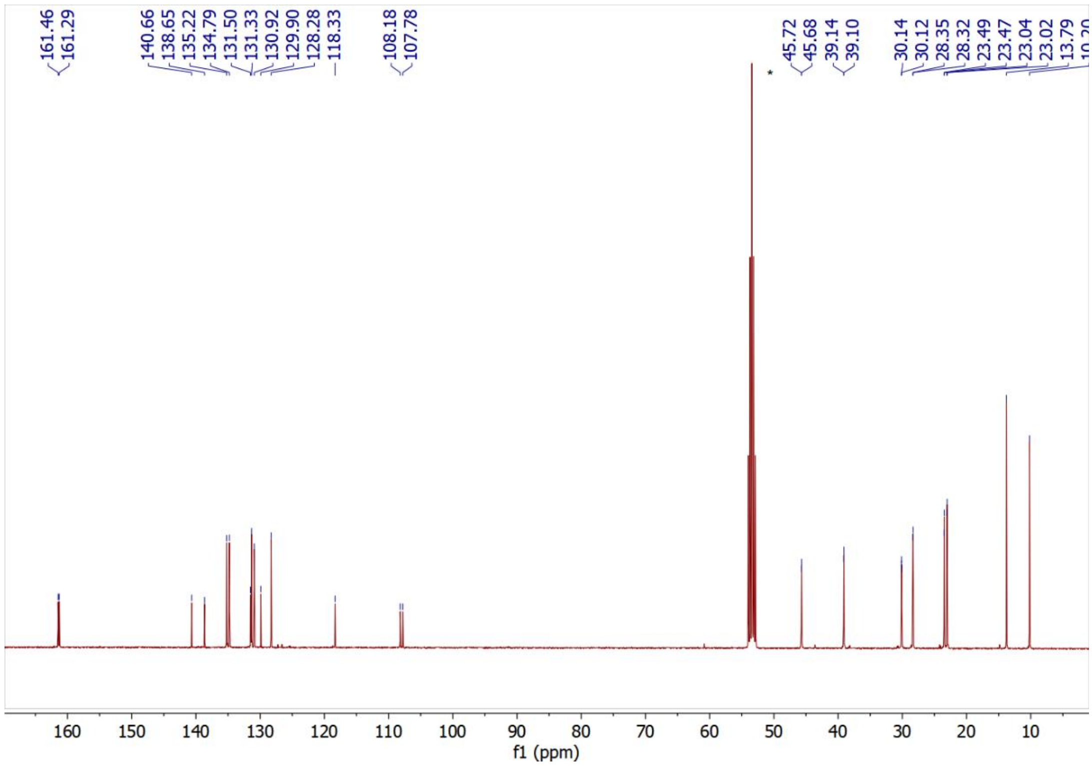

Figure S2. 13C NMR of compound **1** in CD_2Cl_2. Signals relative to solvents are starred.

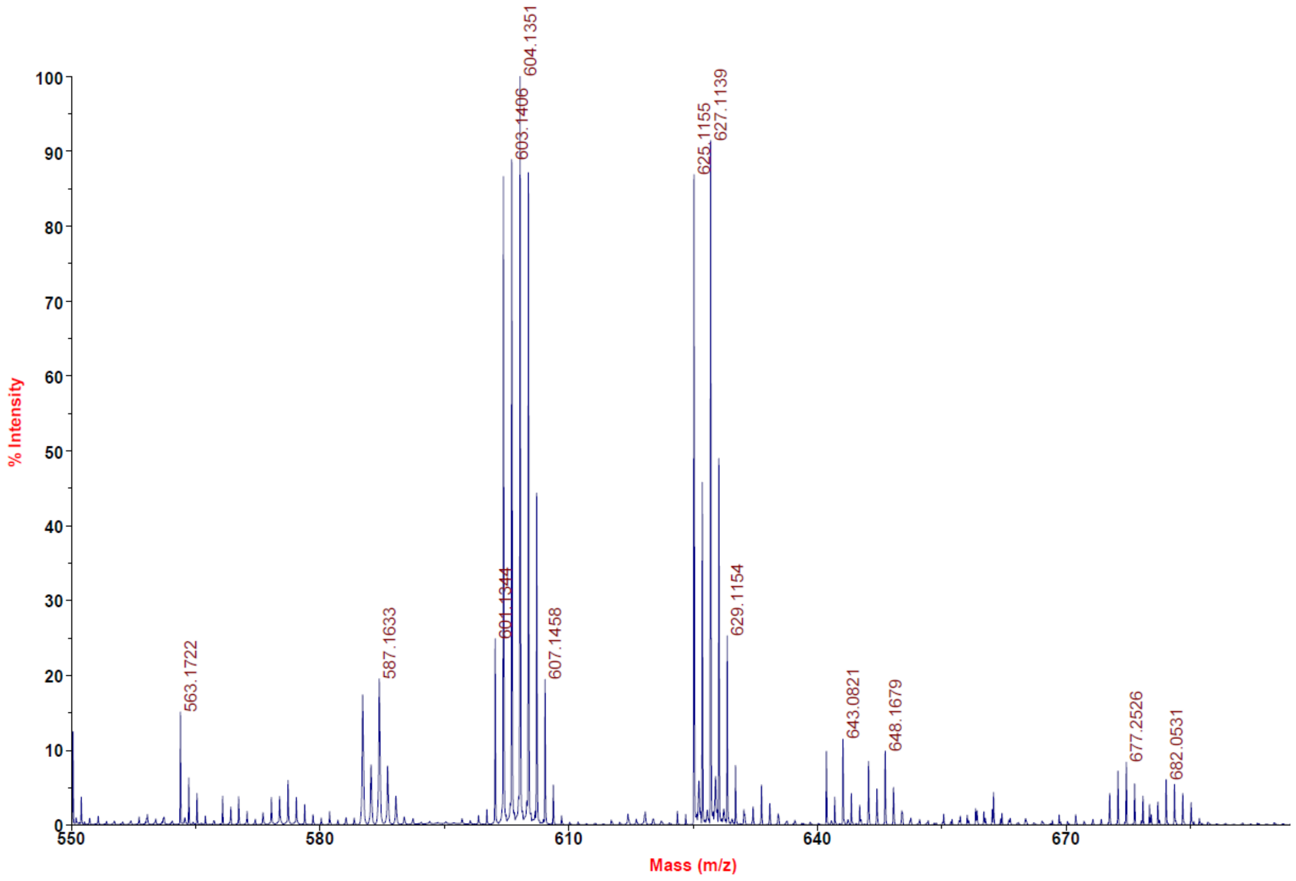

Figure S3. MALDI-TOF mass spectrum of compound 1

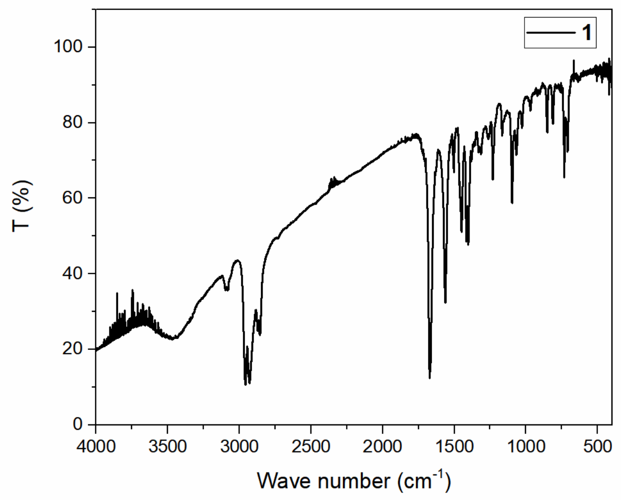

Figure S4. FTIR spectrum of compound **1**.

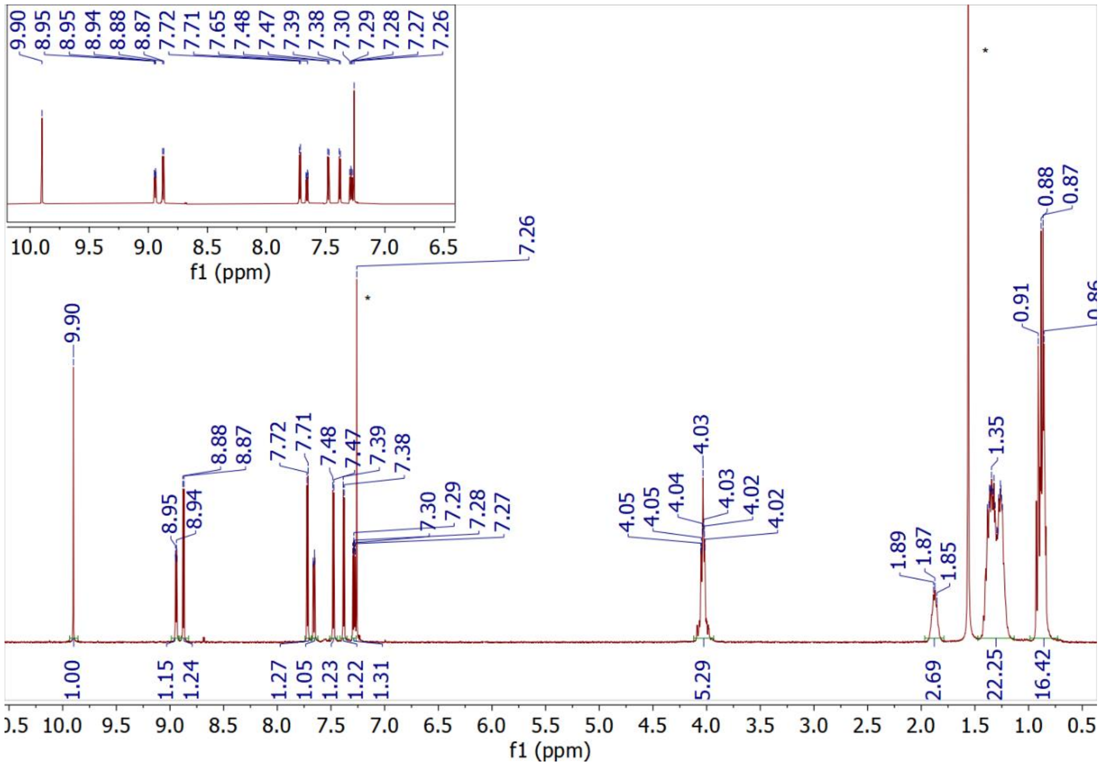

Figure S5. 1H NMR spectrum of **A-CHO** in $CDCl_3$. Signals relative to solvent are starred.

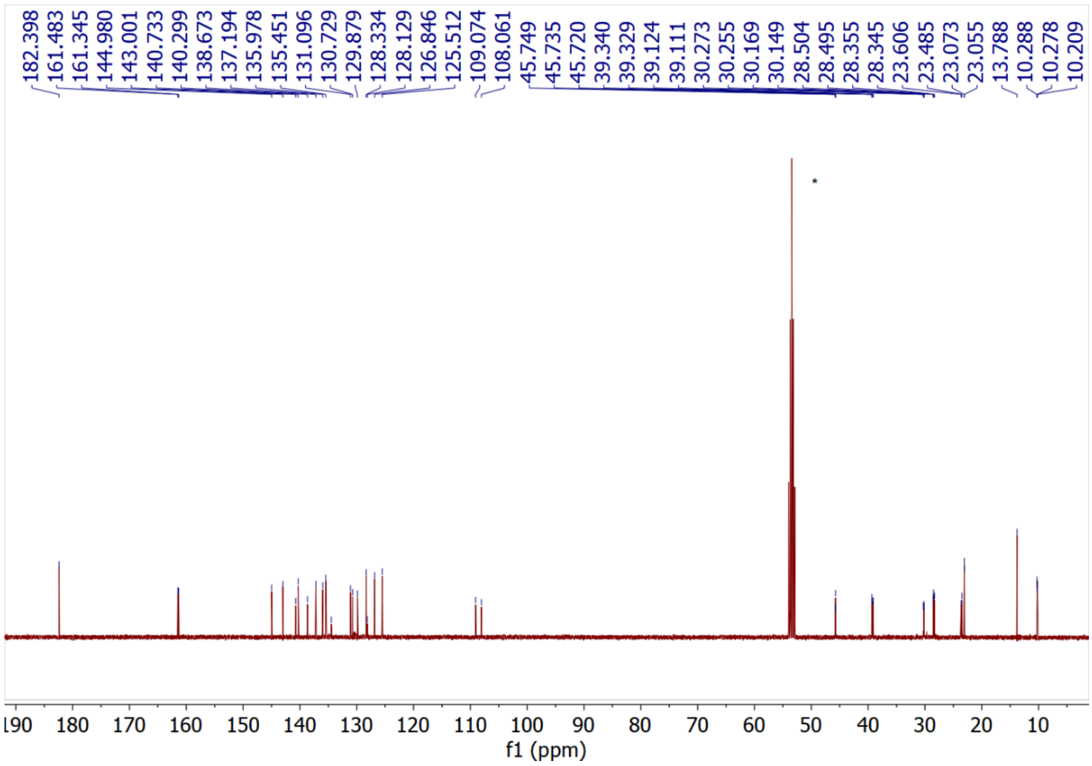

Figure S6. 13C NMR spectrum of **A-CHO** in CD_2Cl_2. Signals relative to solvent are starred.

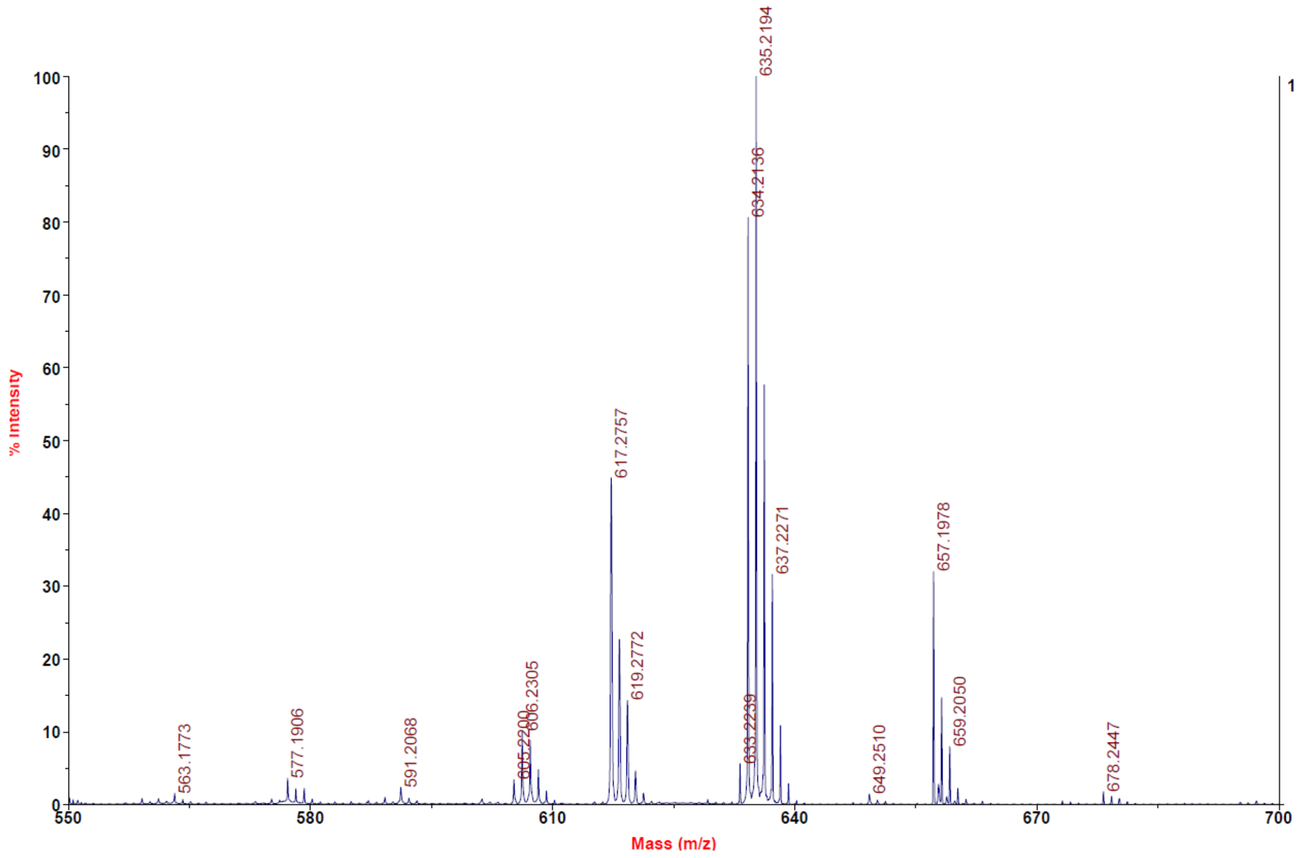

Figure S7. MALDI-TOF mass spectrum of compound **A-CHO**.

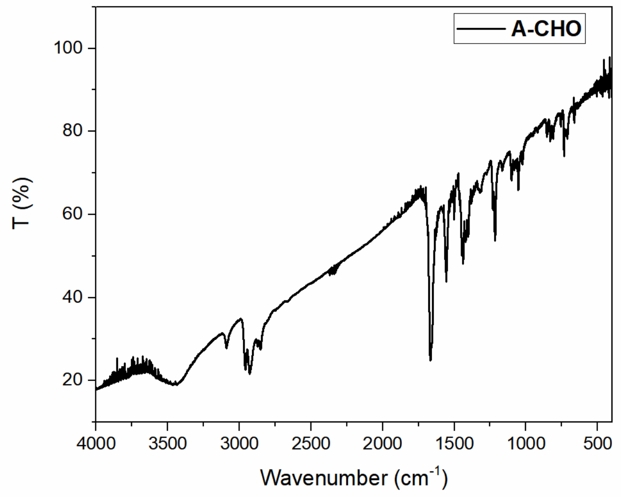

Figure S8. FTIR spectrum of compound **A-CHO**.

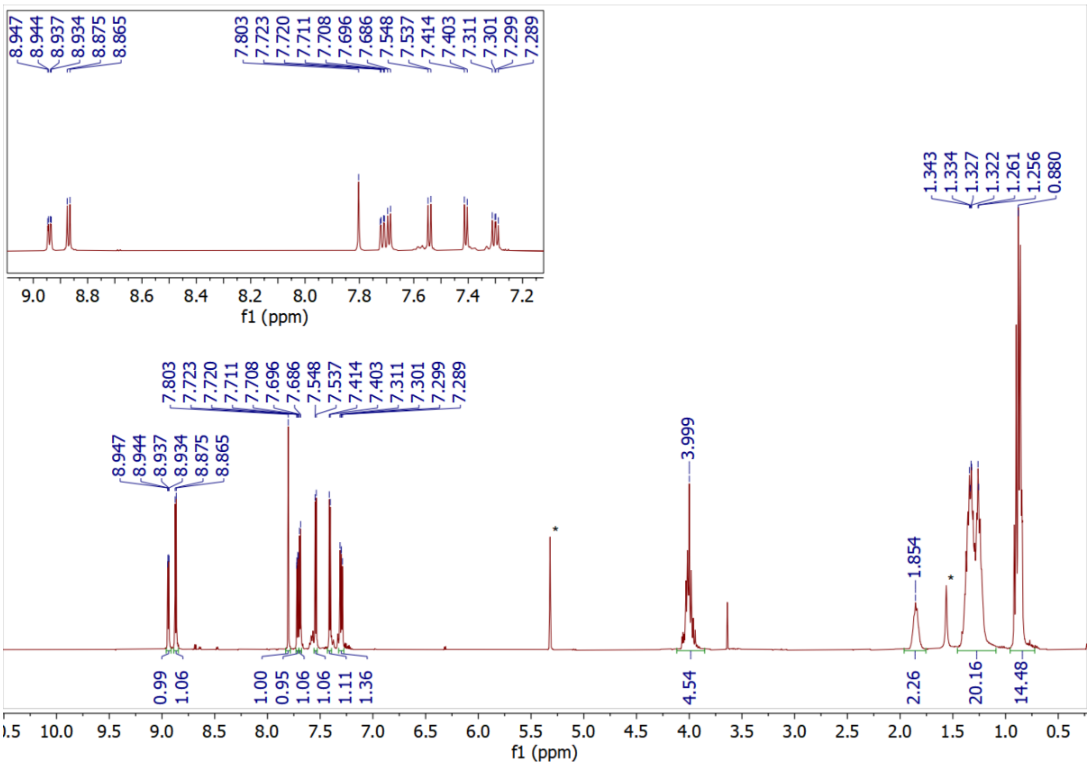

Figure S9. 1H NMR spectrum of **A-DCV** in CD_2Cl_2. Signals relative to solvent are starred.

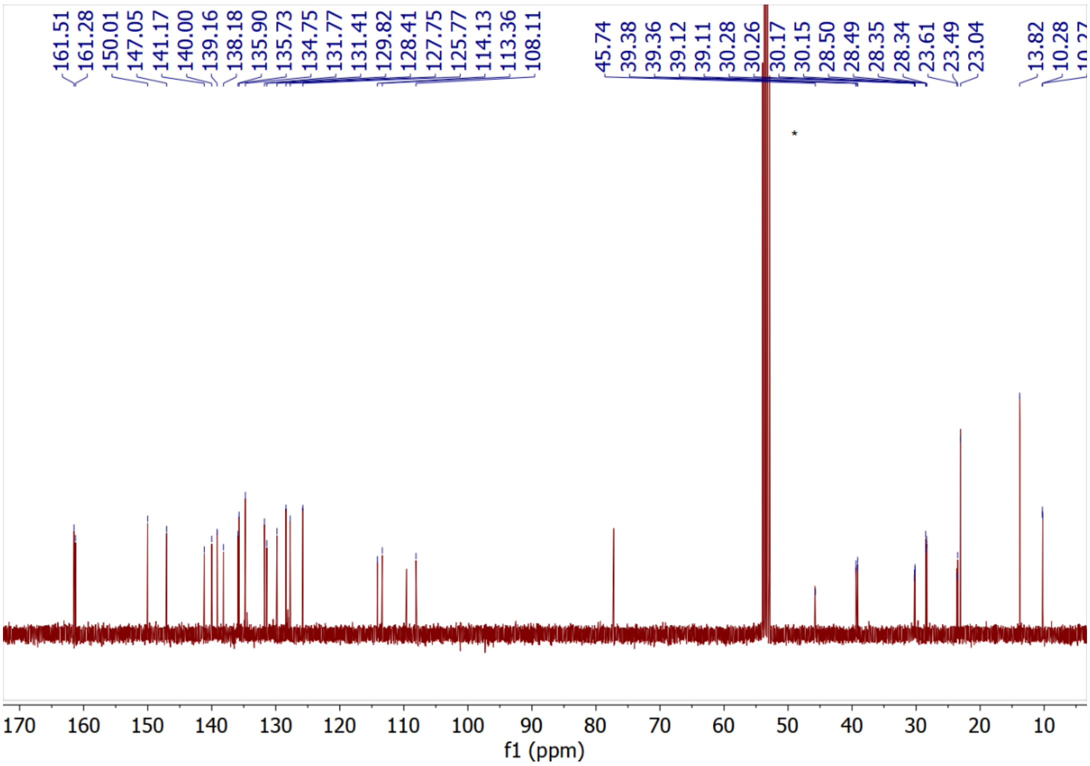

Figure S10. 13C NMR spectrum of **A-DCV** in CD_2Cl_2. Signals relative to solvent are starred.

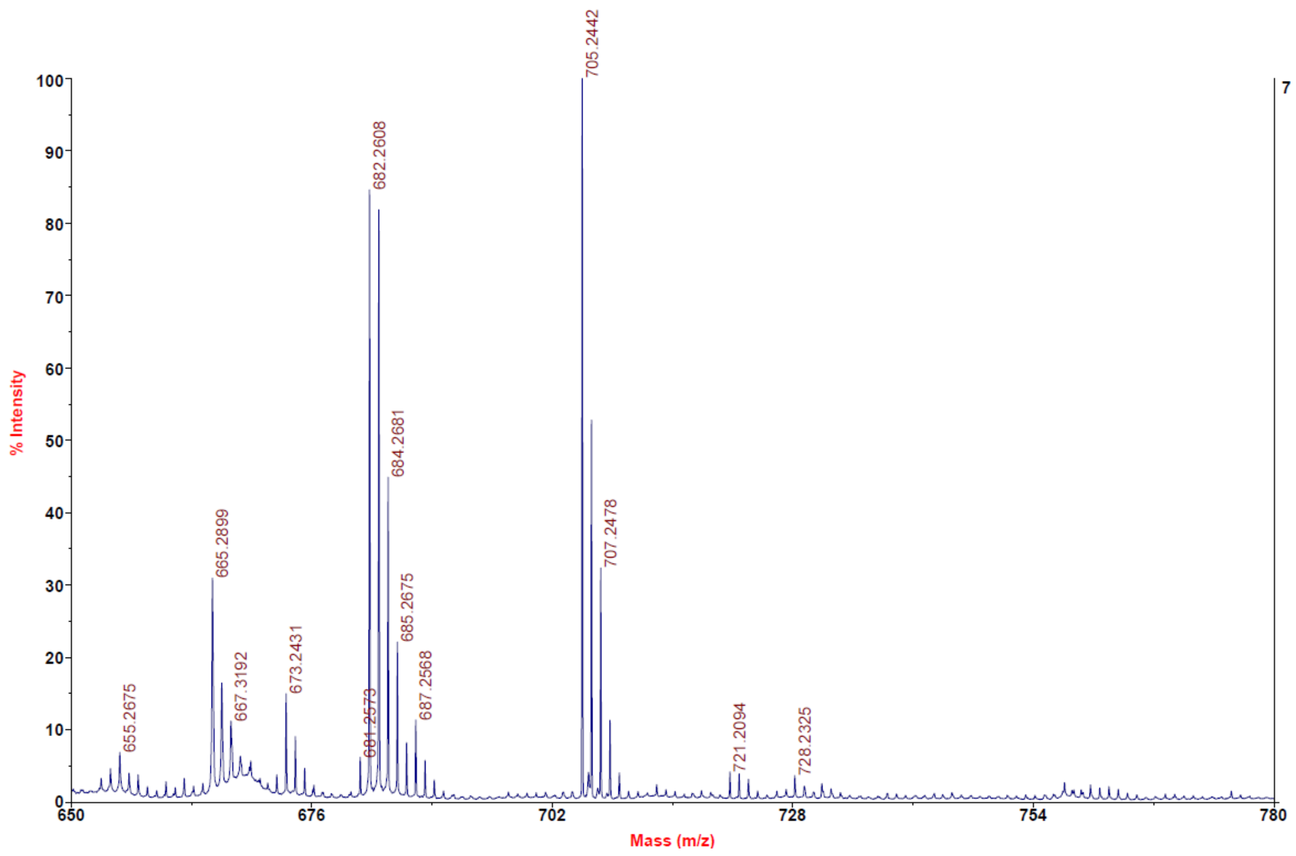

Figure S11. MALDI-TOF mass spectrum of compound **A-DCV**.

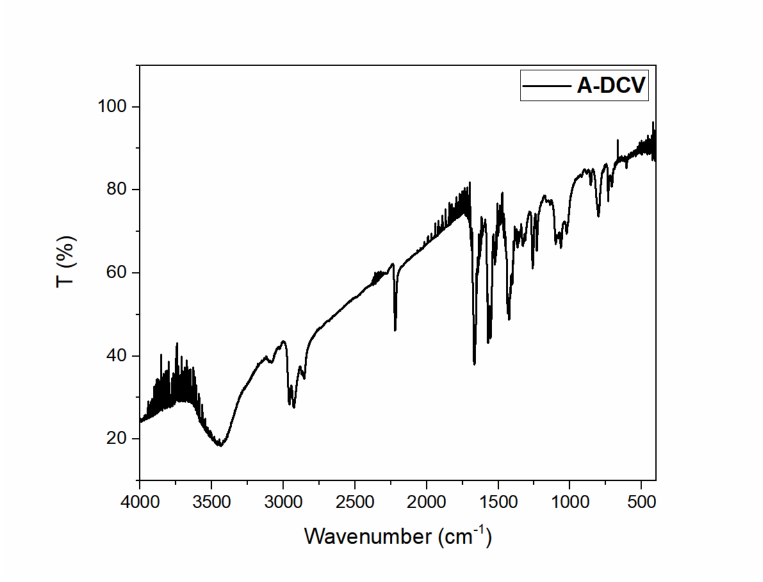

Figure S12. FTIR spectrum of compound **A-DCV**.

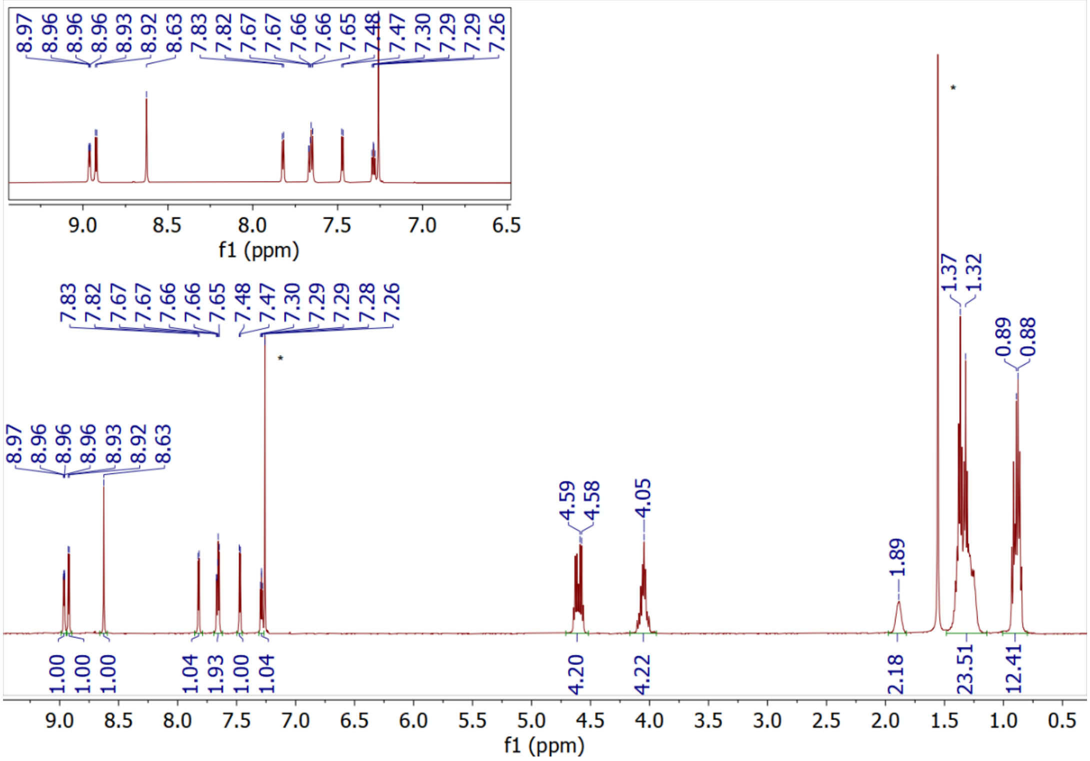

Figure S13. 1H NMR spectrum of **A-TB** in $CDCl_3$. Signals relative to solvent are starred.

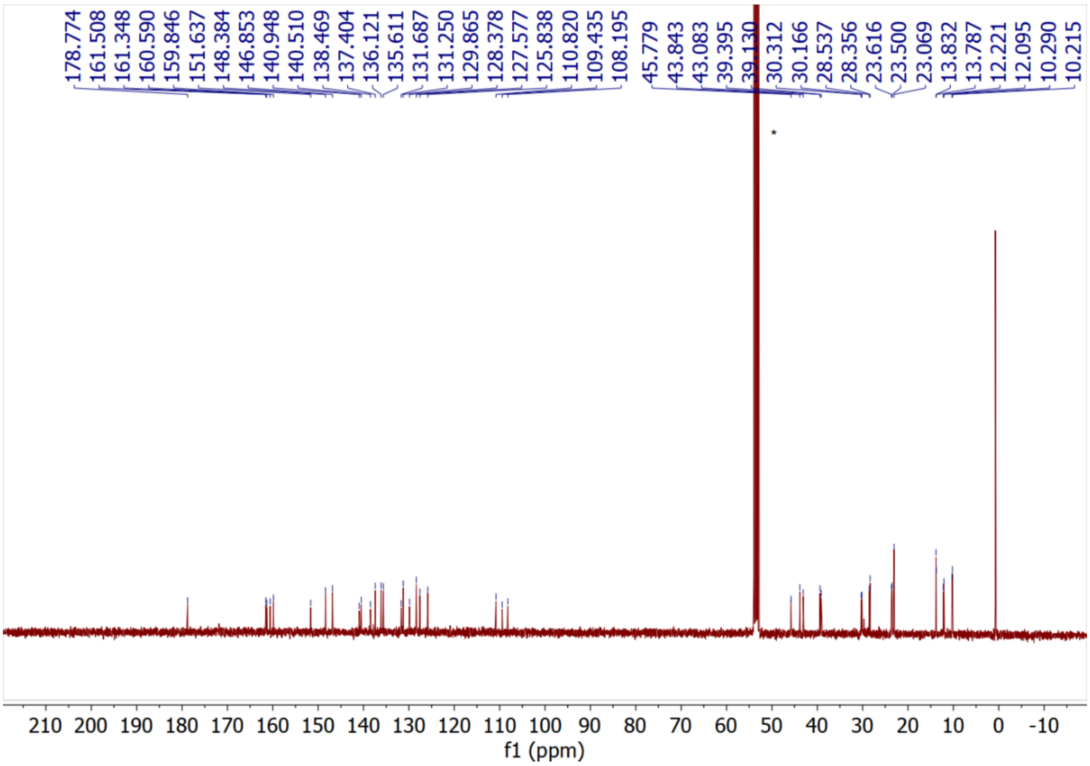

Figure S14. 13C NMR spectrum of **A-TB** in $CDCl_3$. Signals relative to solvent are starred.

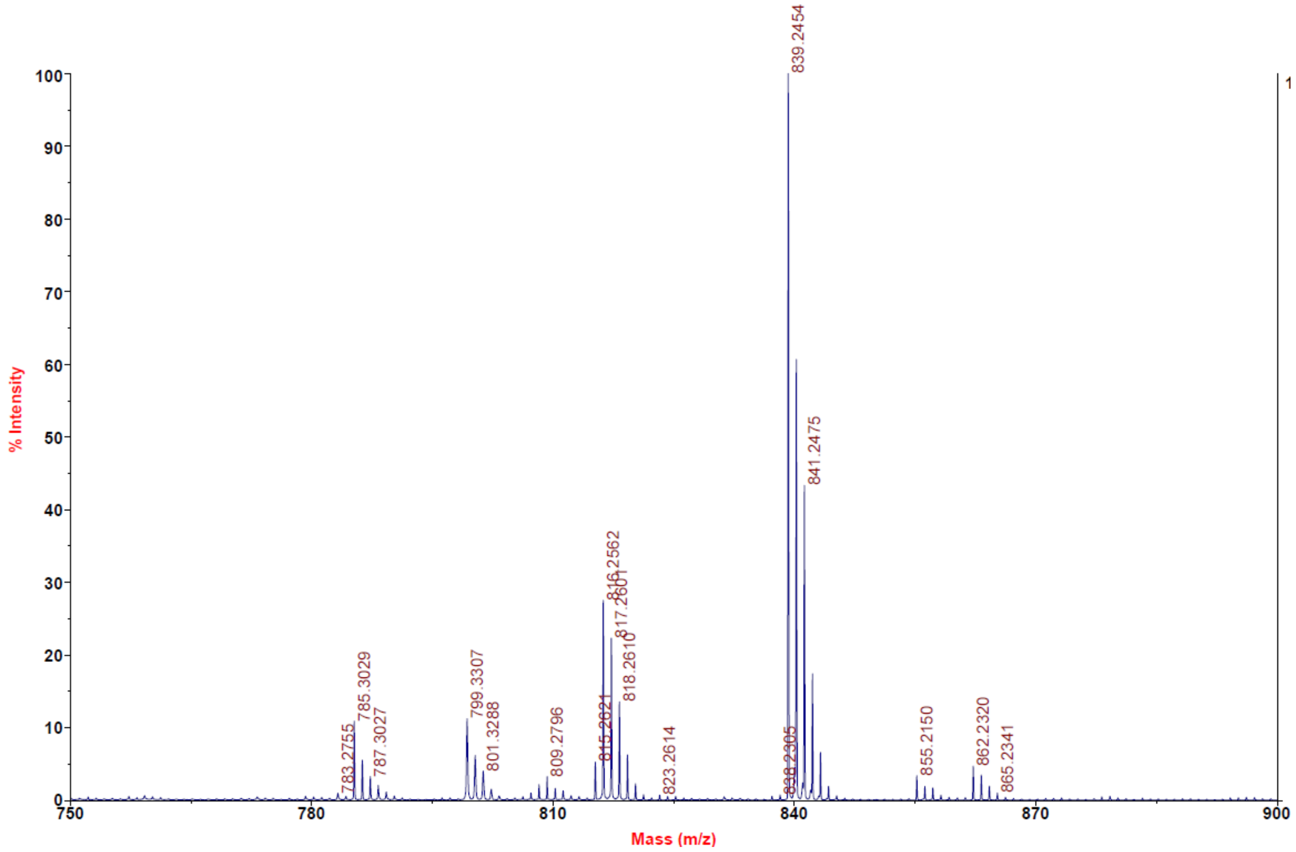

Figure S15. MALDI-TOF mass spectrum of compound **A-TB**.

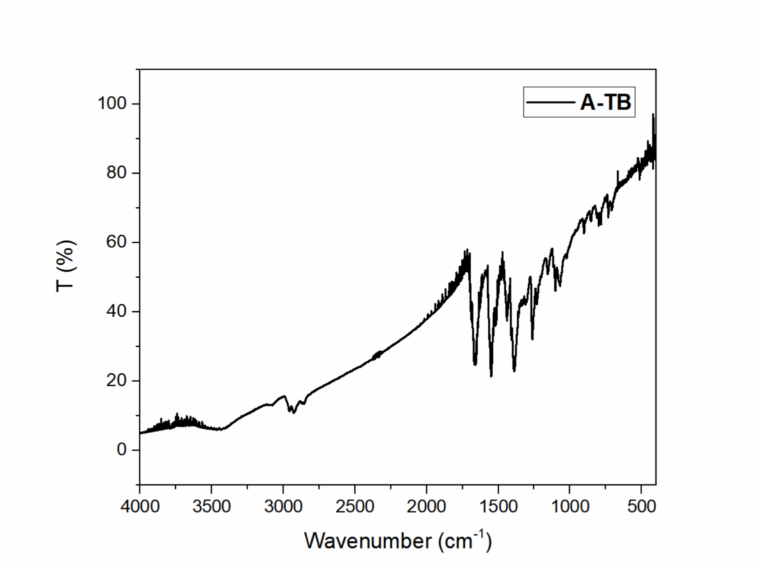

Figure S16. FTIR spectrum of compound **A-TB**.

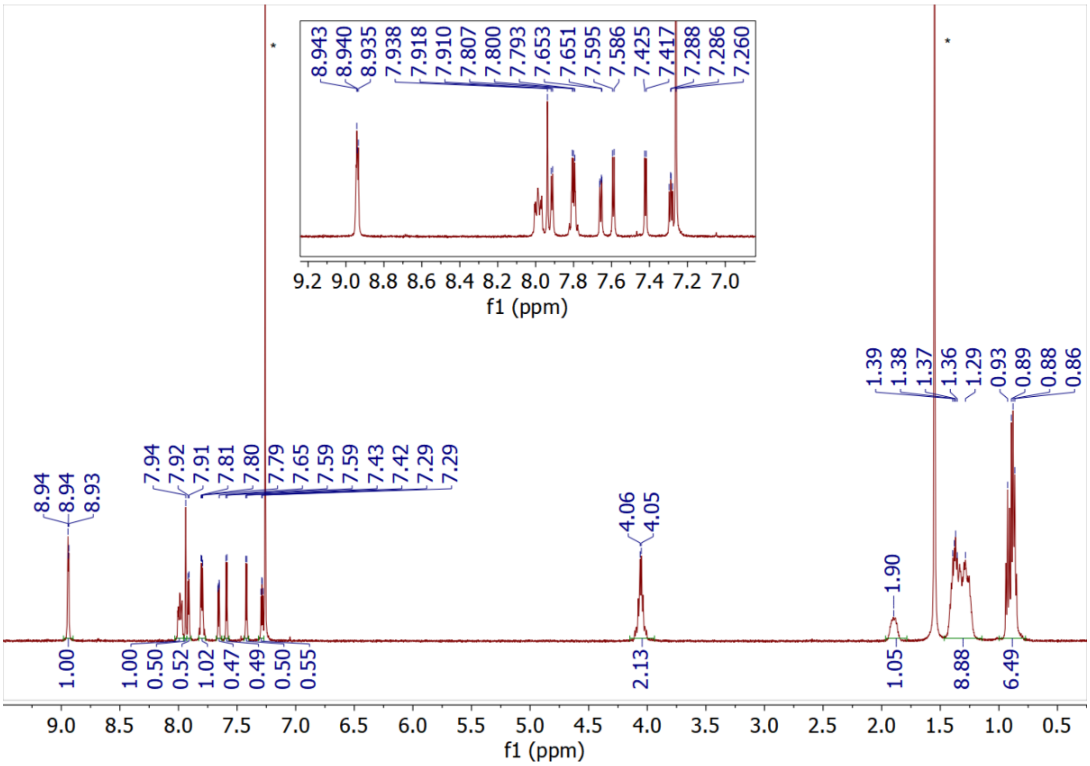

Figure S17. 1H NMR spectrum of **A-ID** in $CDCl_3$. Signals relative to solvent are starred.

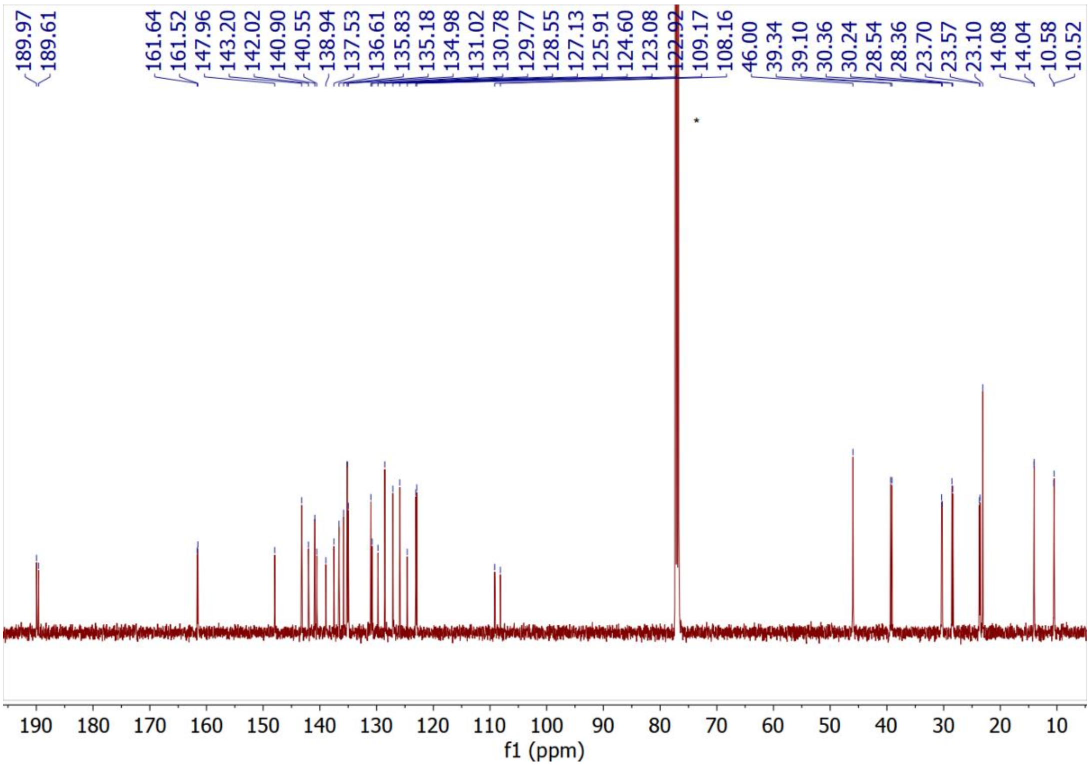

Figure S18. 13C NMR spectrum of **A-ID** in $CDCl_3$. Signals relative to solvent are starred.

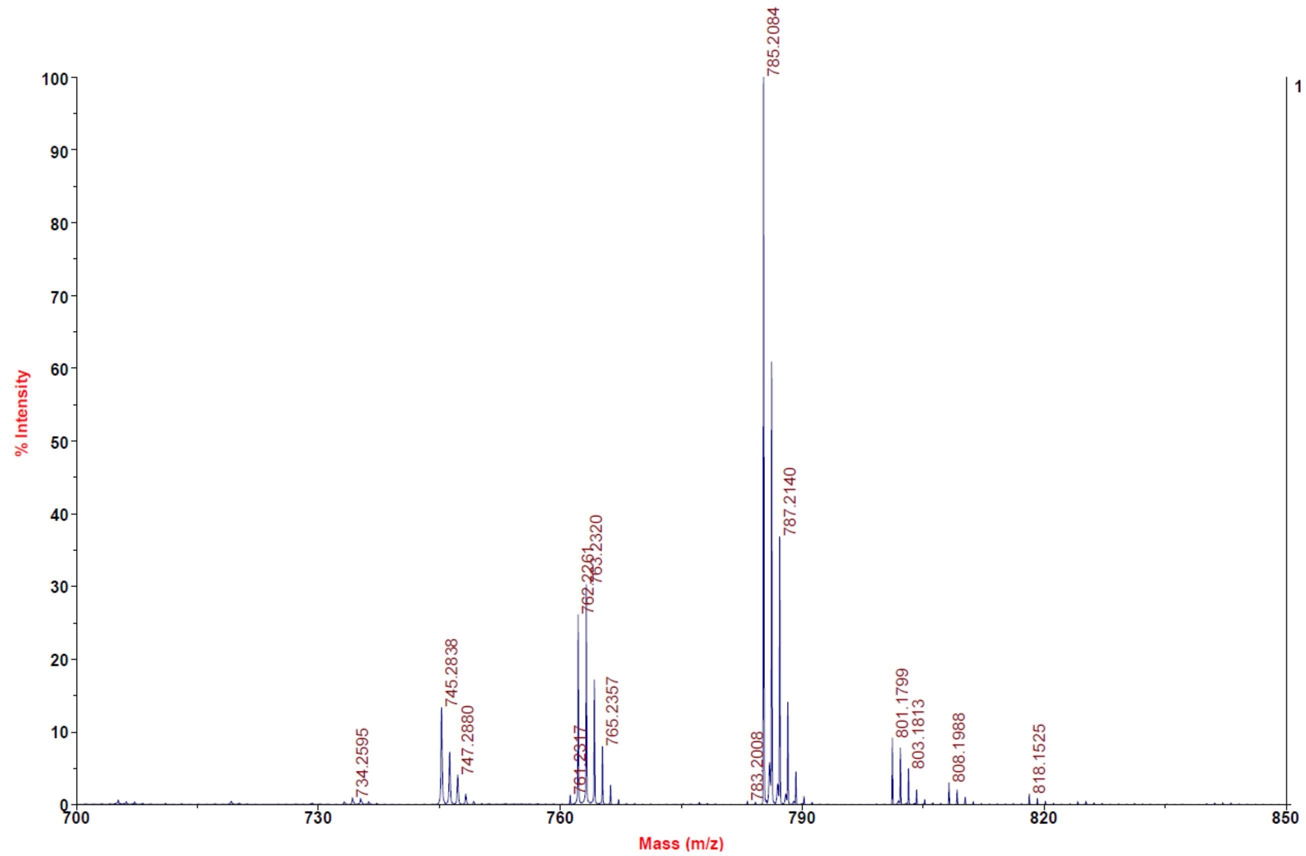

Figure S19. MALDI-TOF mass spectrum of compound **A-ID**.

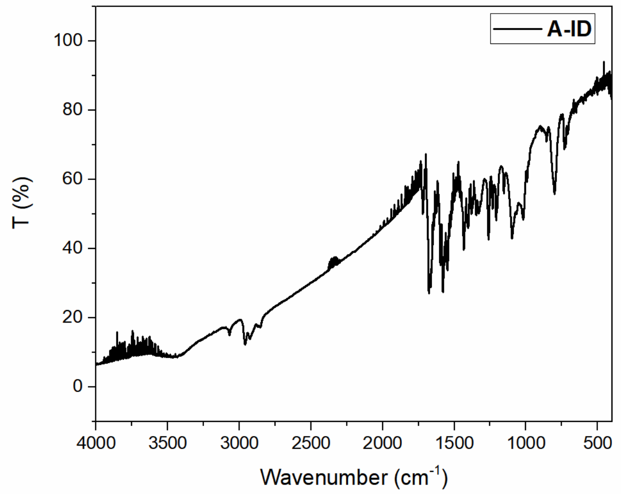

Figure S20. FTIR spectrum of compound **A-ID**.

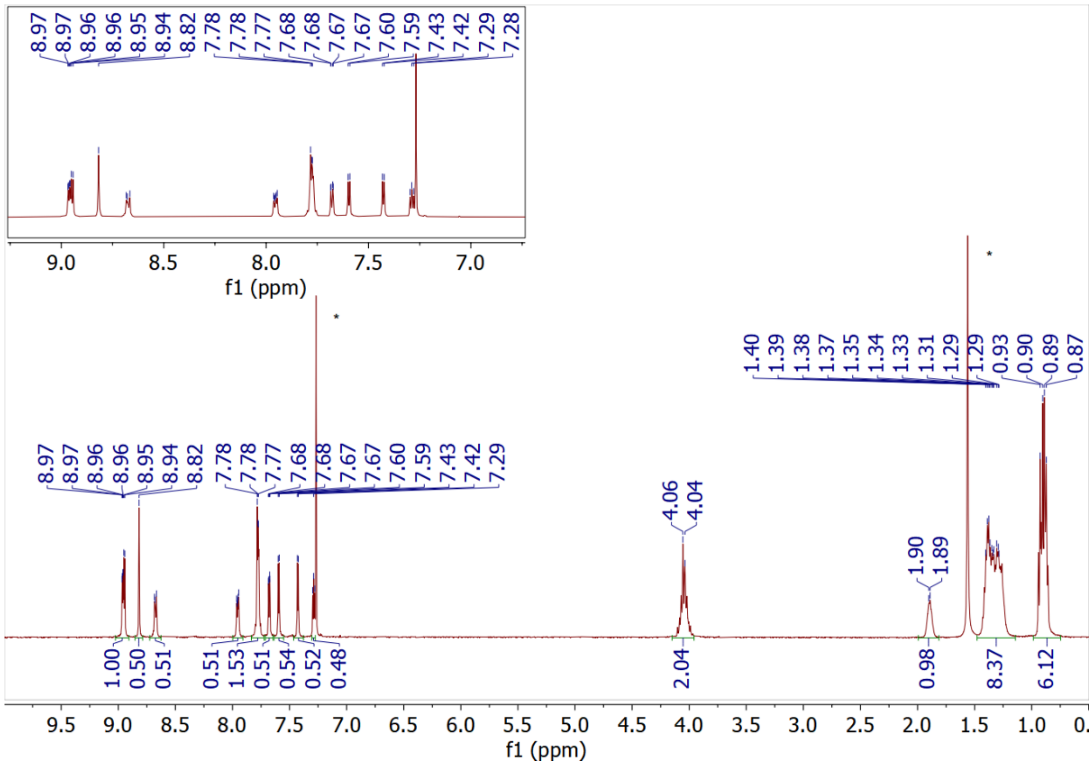

Figure S21. 1H NMR spectrum of **A-IDM** in $CDCl_3$. Signals relative to solvent are starred.

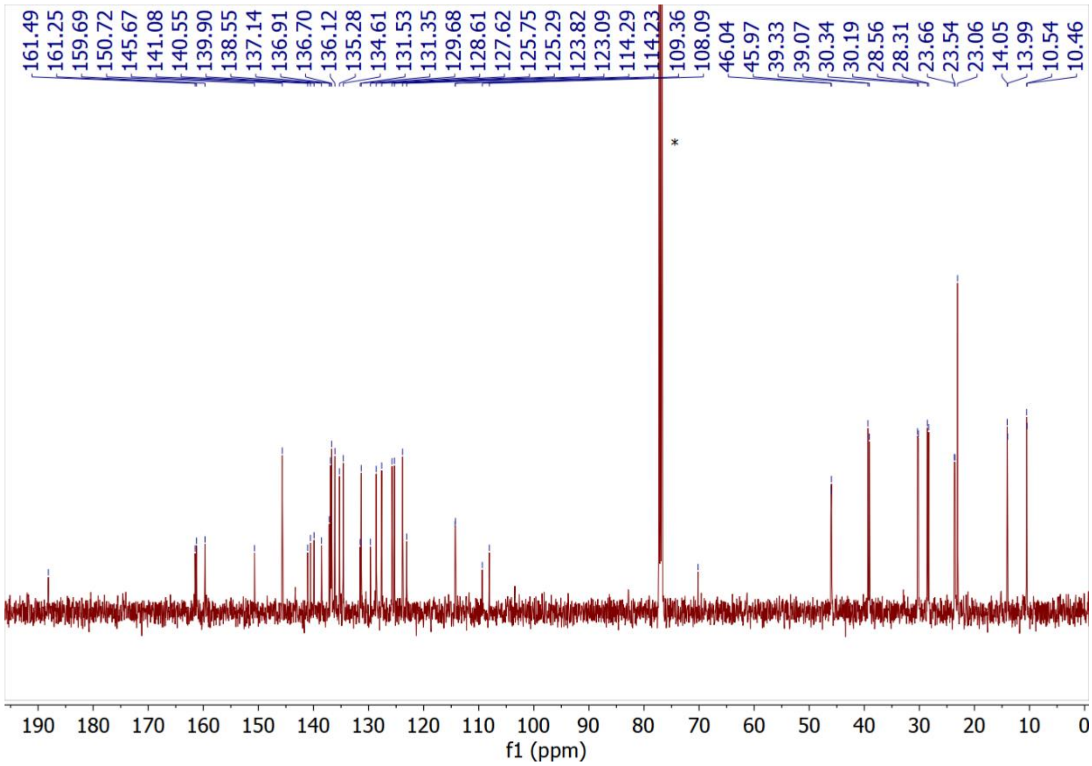

Figure S22. 13C NMR spectrum of **A-IDM** in $CDCl_3$. Signals relative to solvent are starred.

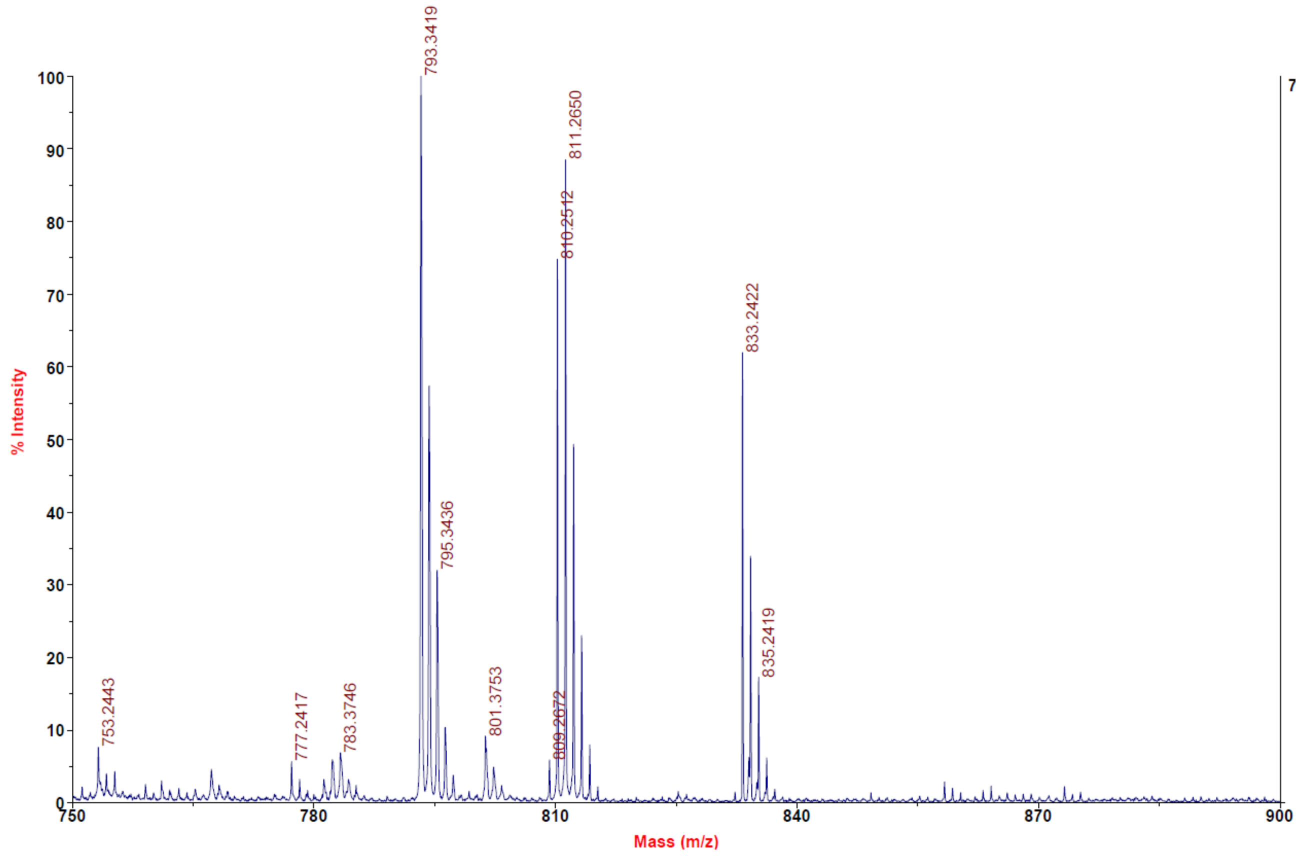

Figure S23. MALDI-TOF mass spectrum of compound **A-IDM**.

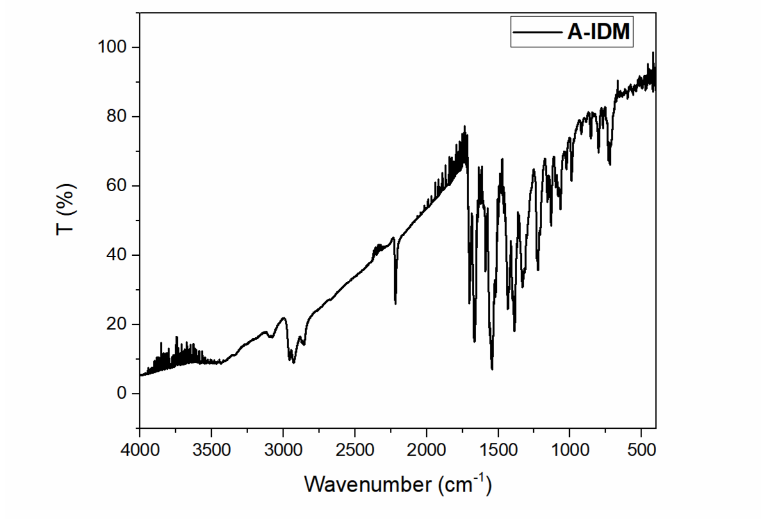

Figure S24. FTIR spectrum of compound **A-IDM**.

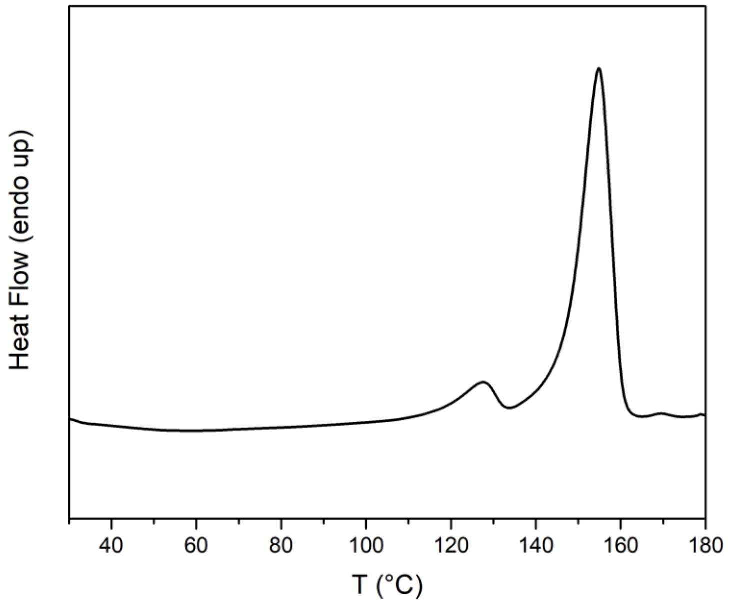

Figure S25. DSC trace of compound **A-DCV**.

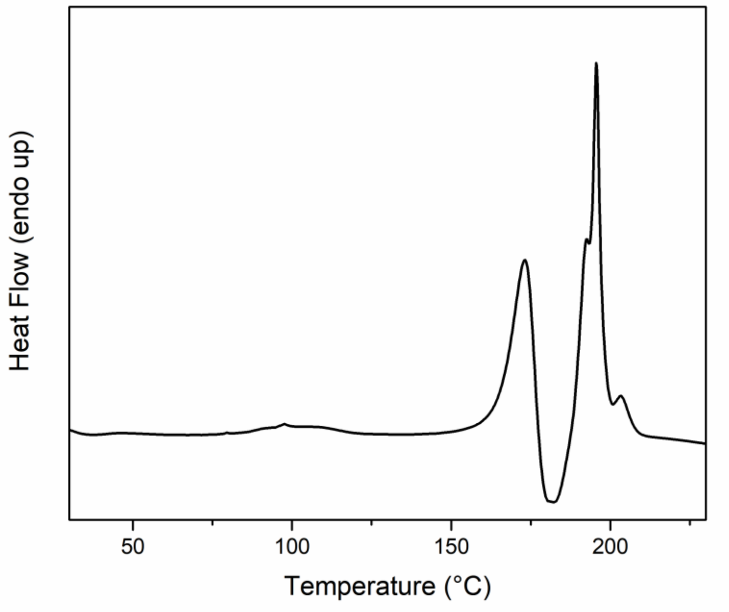

Figure S26. DSC trace of compound **A-TB**.

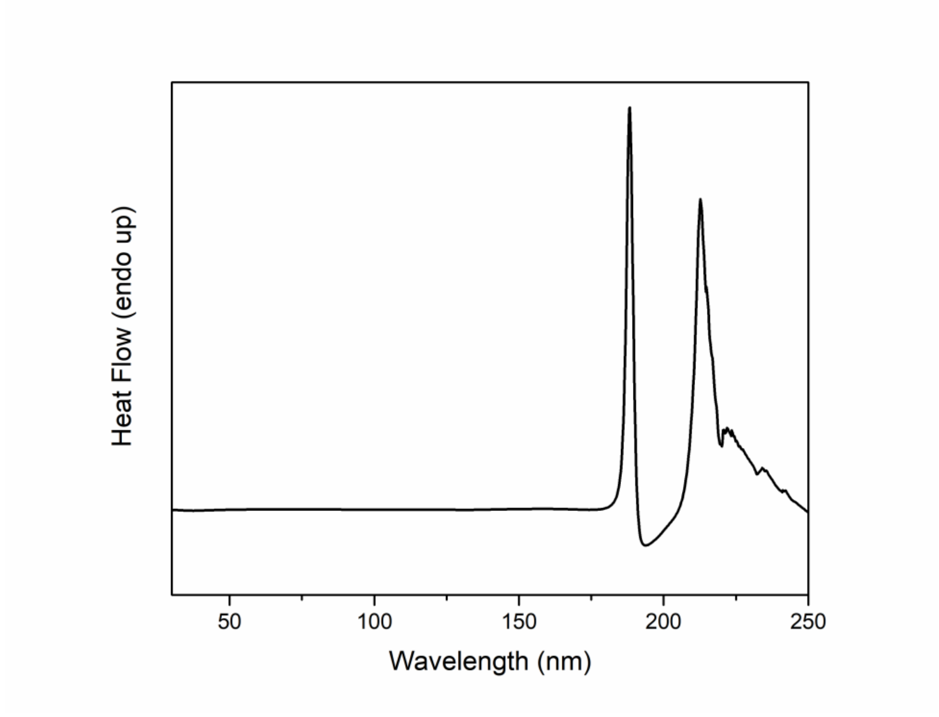

Figure S27. DSC trace of compound **A-ID**.

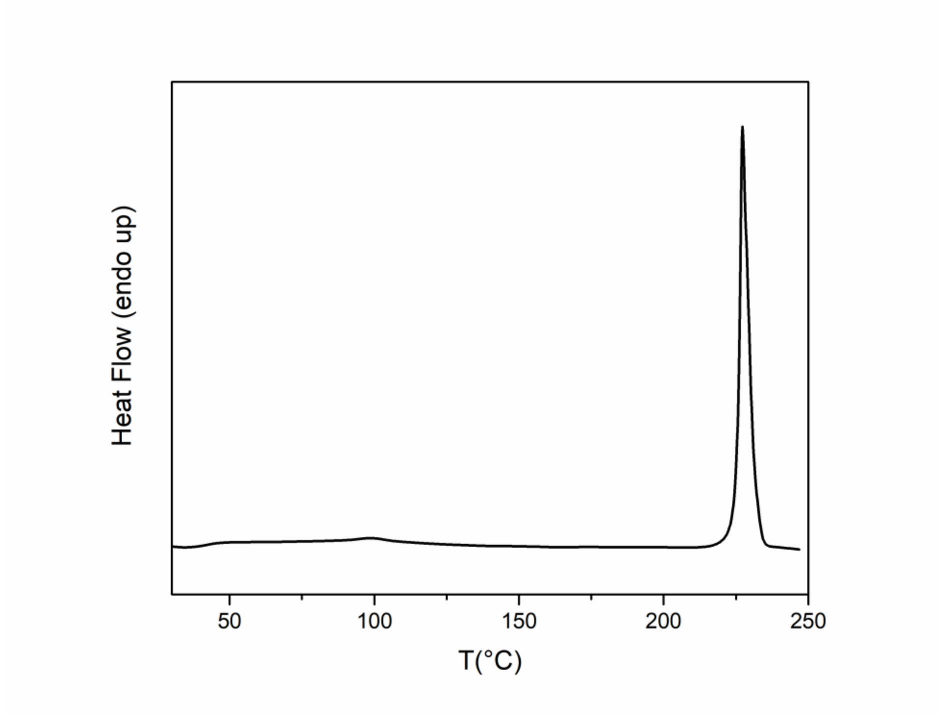

Figure S28. DSC trace of compound **A-IDM**.

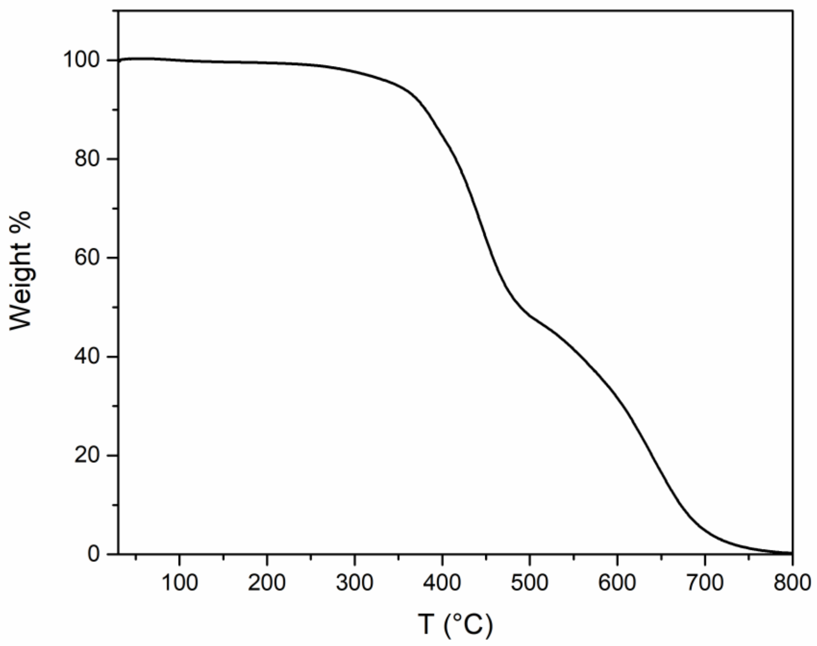

Figure S29. TGA graph of compound **A-DCV**.

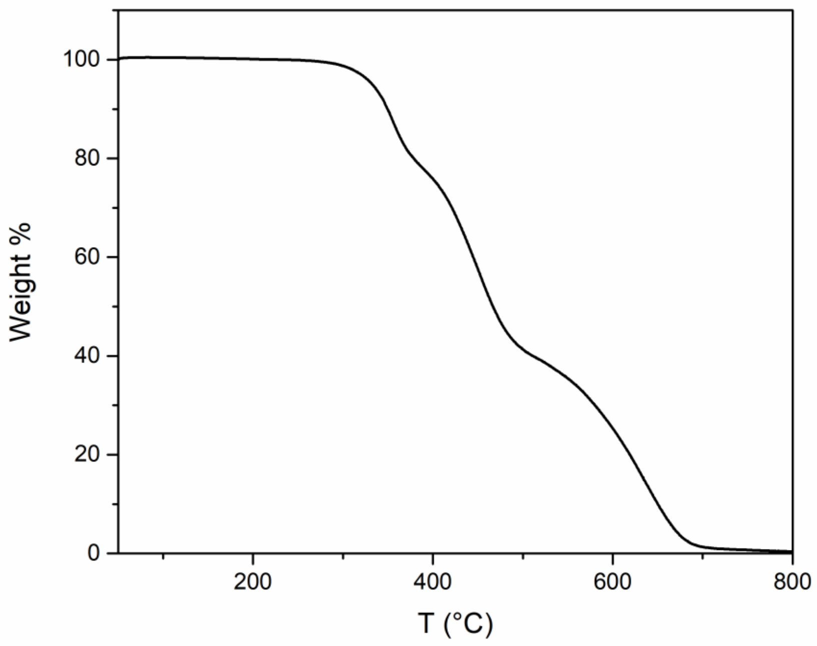

Figure S30. TGA graph of compound **A-TB**.

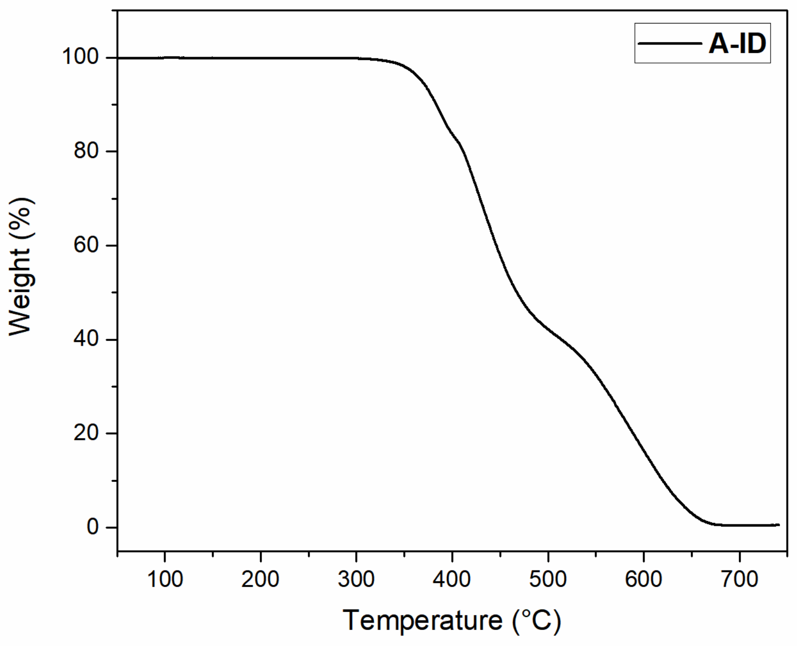

Figure S31. TGA graph of compound **A-ID**.

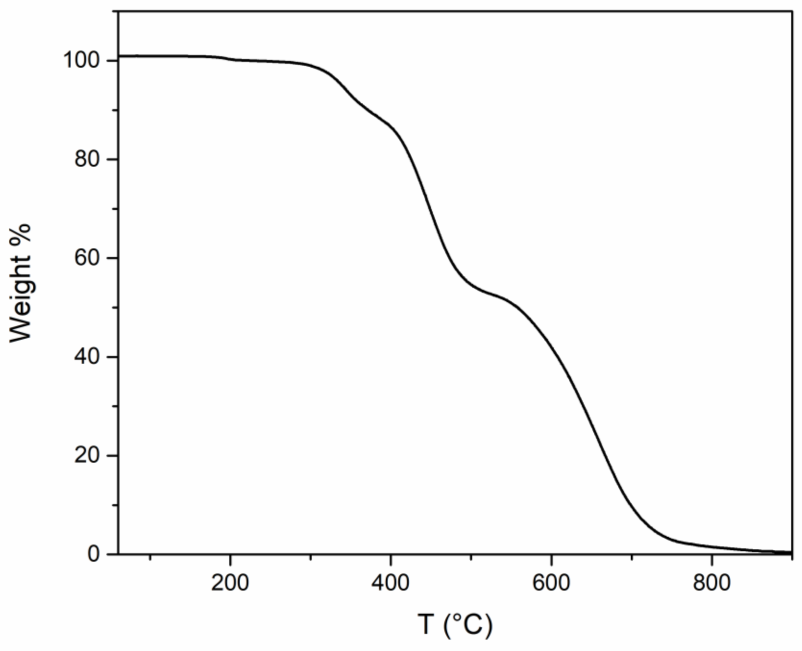

Figure S32. TGA graph of compound **A-IDM**.

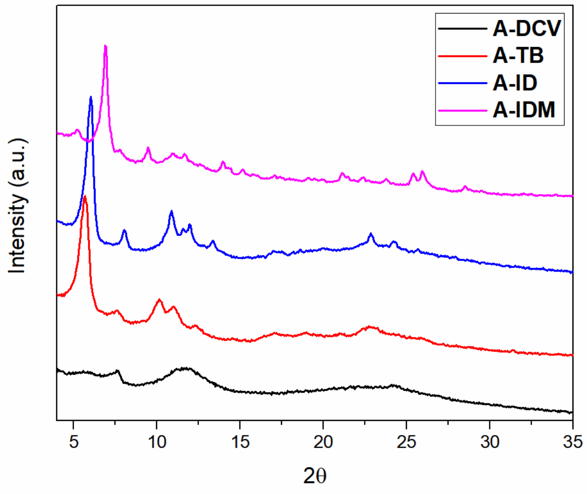

Figure S33. XRD spectra on drop-casted films of the dyes.

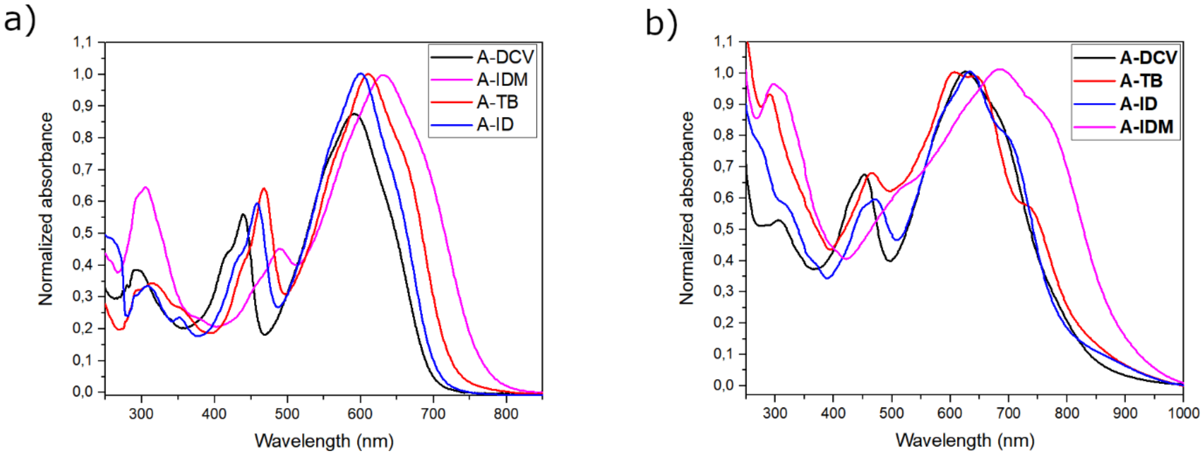

Figure S34. a) Optical absorption spectra of the dyes in THF solution ($1 \cdot 10^{-5}$ M); b) thin films of the dyes spin coated by THF solution (b).

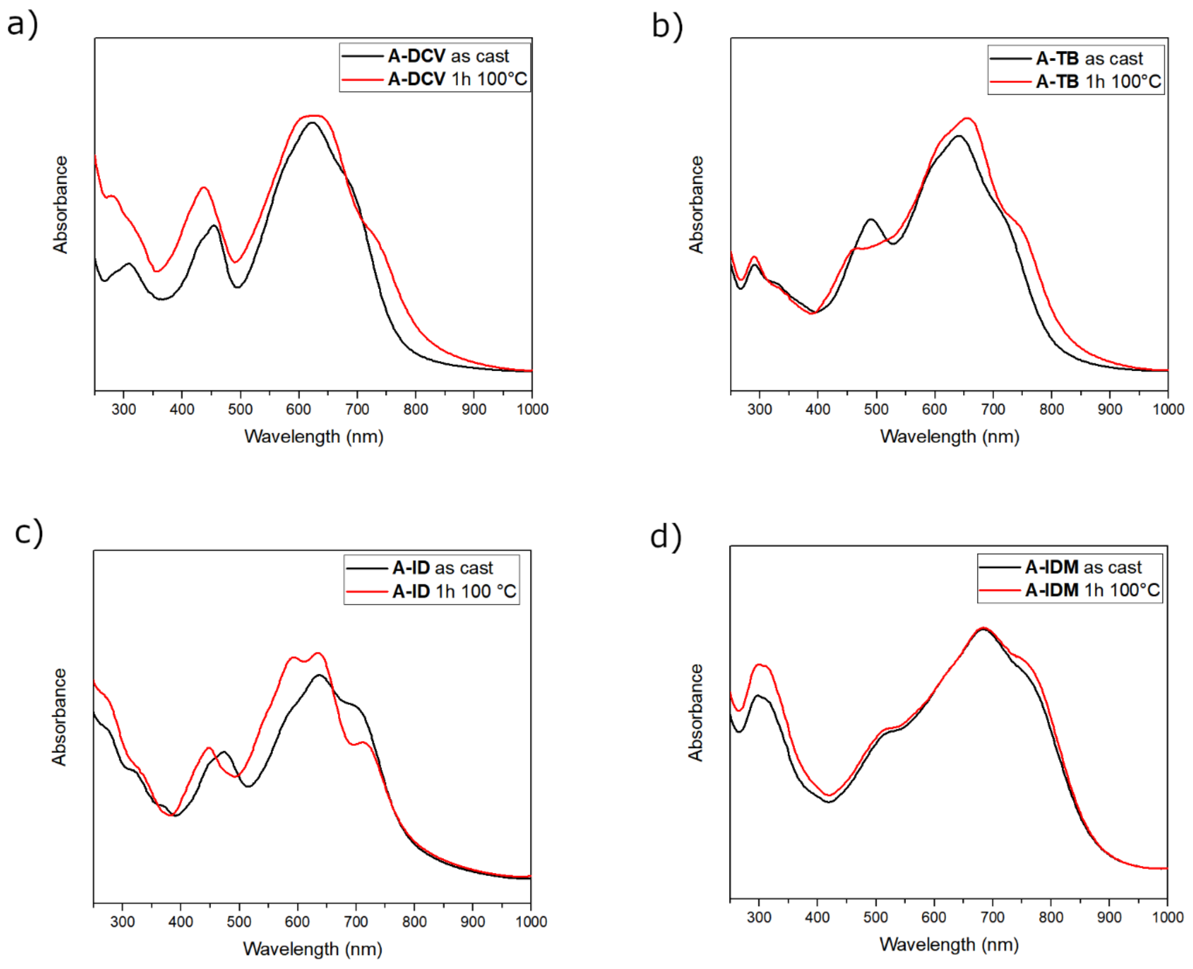

Figure S35. Effect of annealing on optical spectra of a) **A-DCV**, B) **A-TB**, c) **A-ID** and d) **A-IDM**.

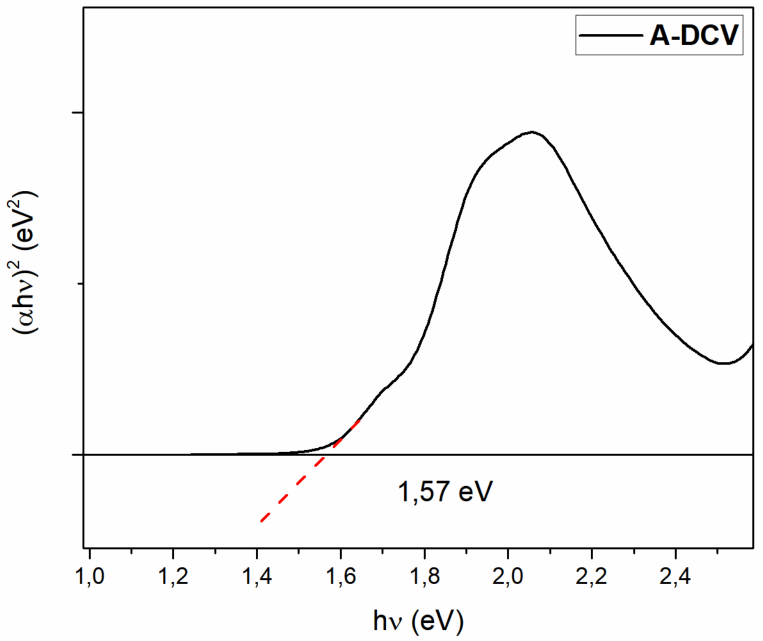

Figure S36. Extrapolation of optical bandgap for **A-DCV** using Tauc plot methodology.

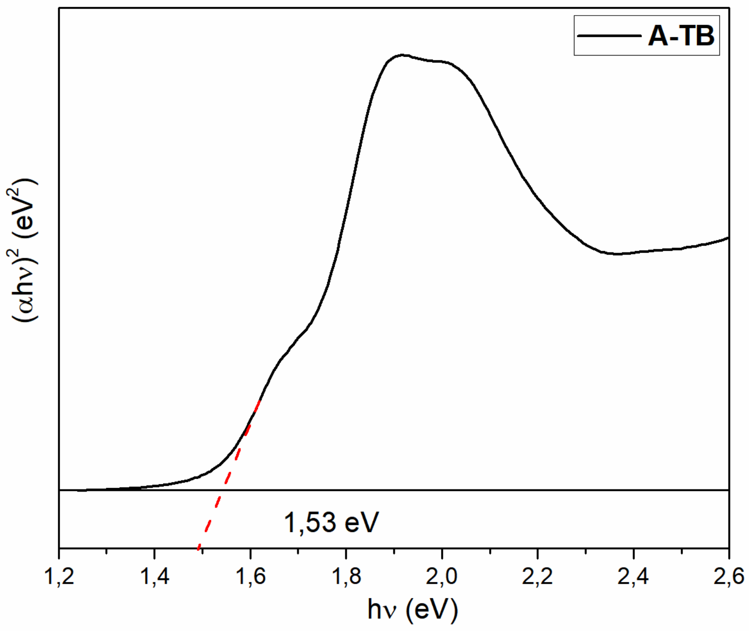

Figure S37. Extrapolation of optical bandgap for **A-TB** using Tauc plot methodology.

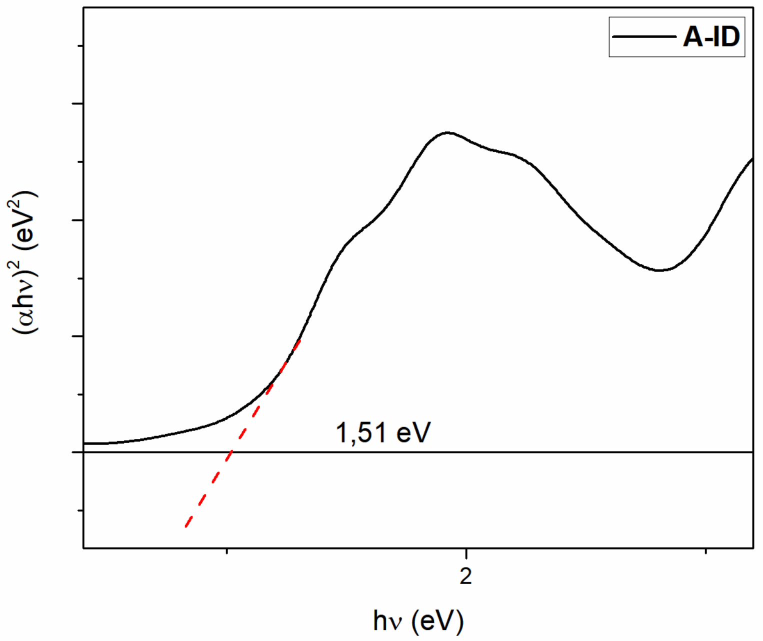

Figure S38. Extrapolation of optical bandgap for **A-ID** using Tauc plot methodology.

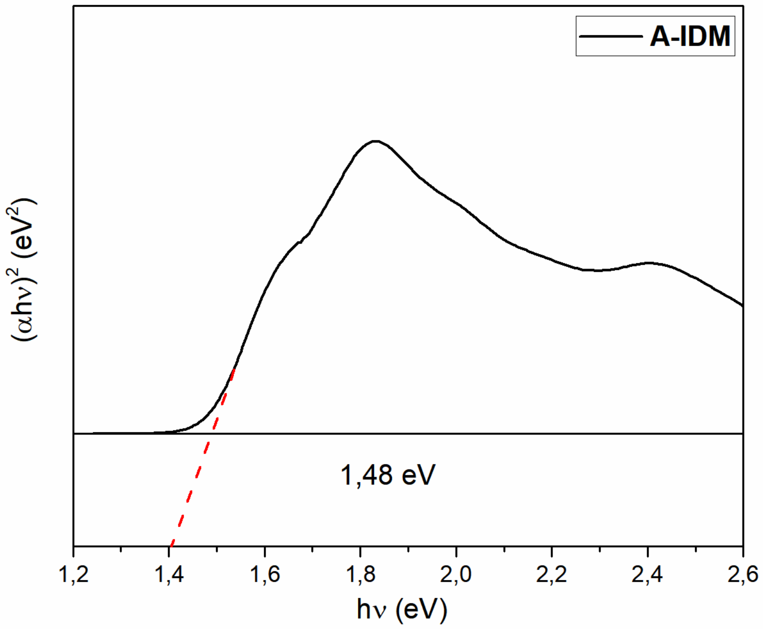

Figure S39. Extrapolation of optical bandgap for **A-IDM** using Tauc plot methodology.

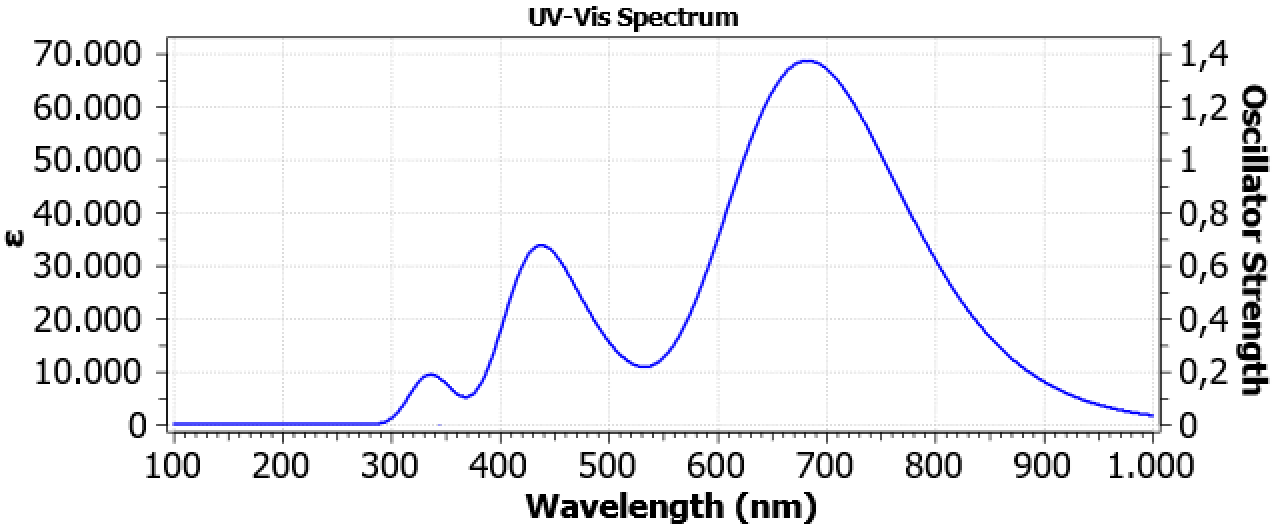

Figure S40. Predicted UV spectrum of **A-DCV**.

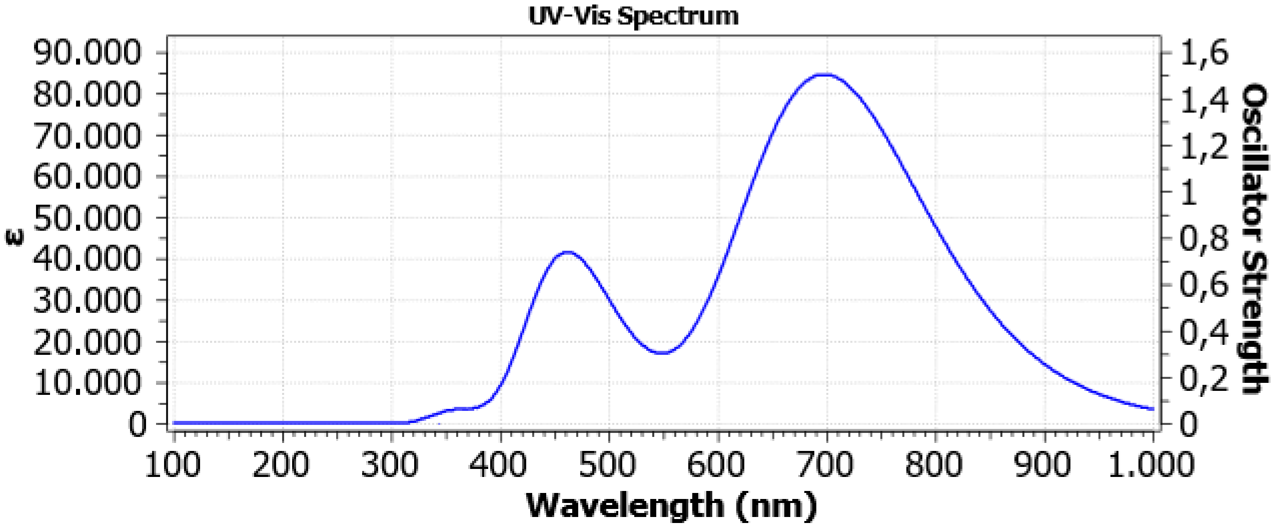

Figure S41. Predicted UV spectrum of **A-TB**.

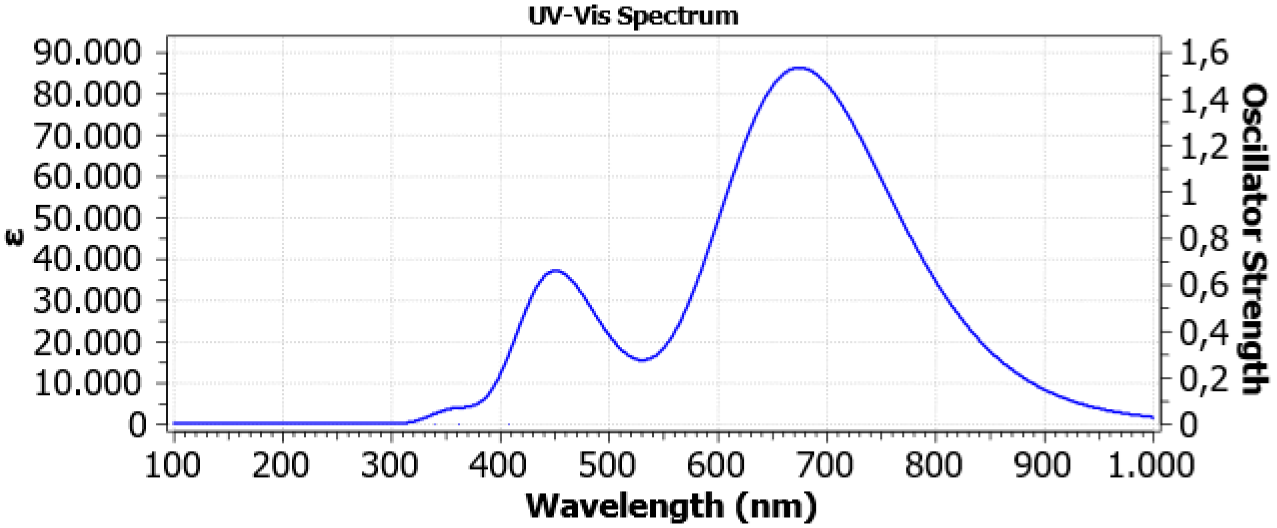

Figure S42. Predicted UV spectrum of **A-ID**.

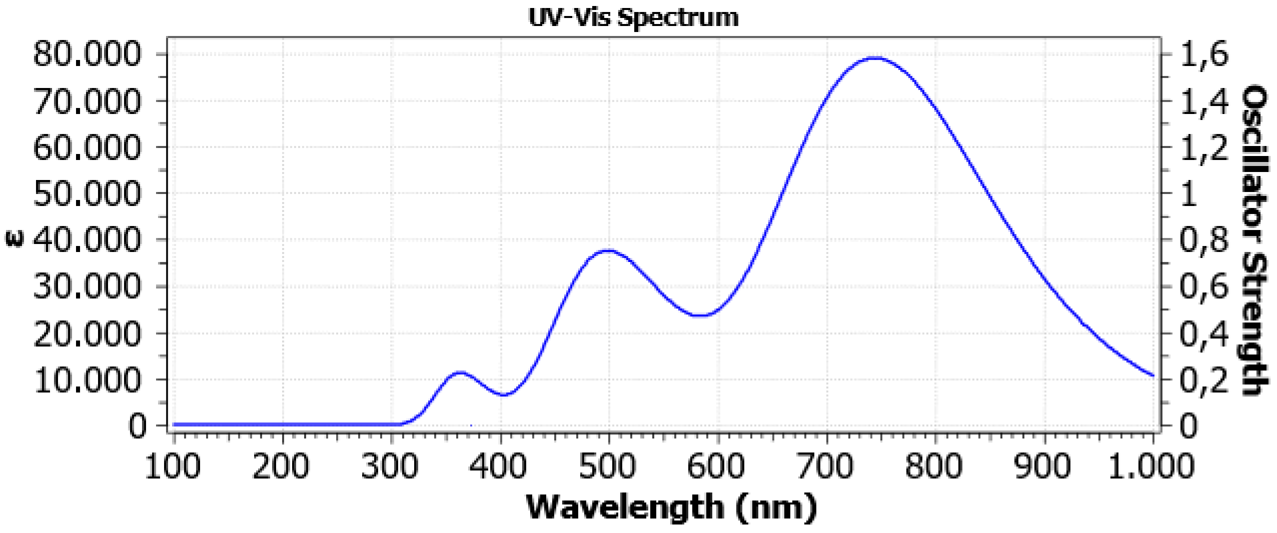

Figure S43. Predicted UV spectrum of **A-IDM**.

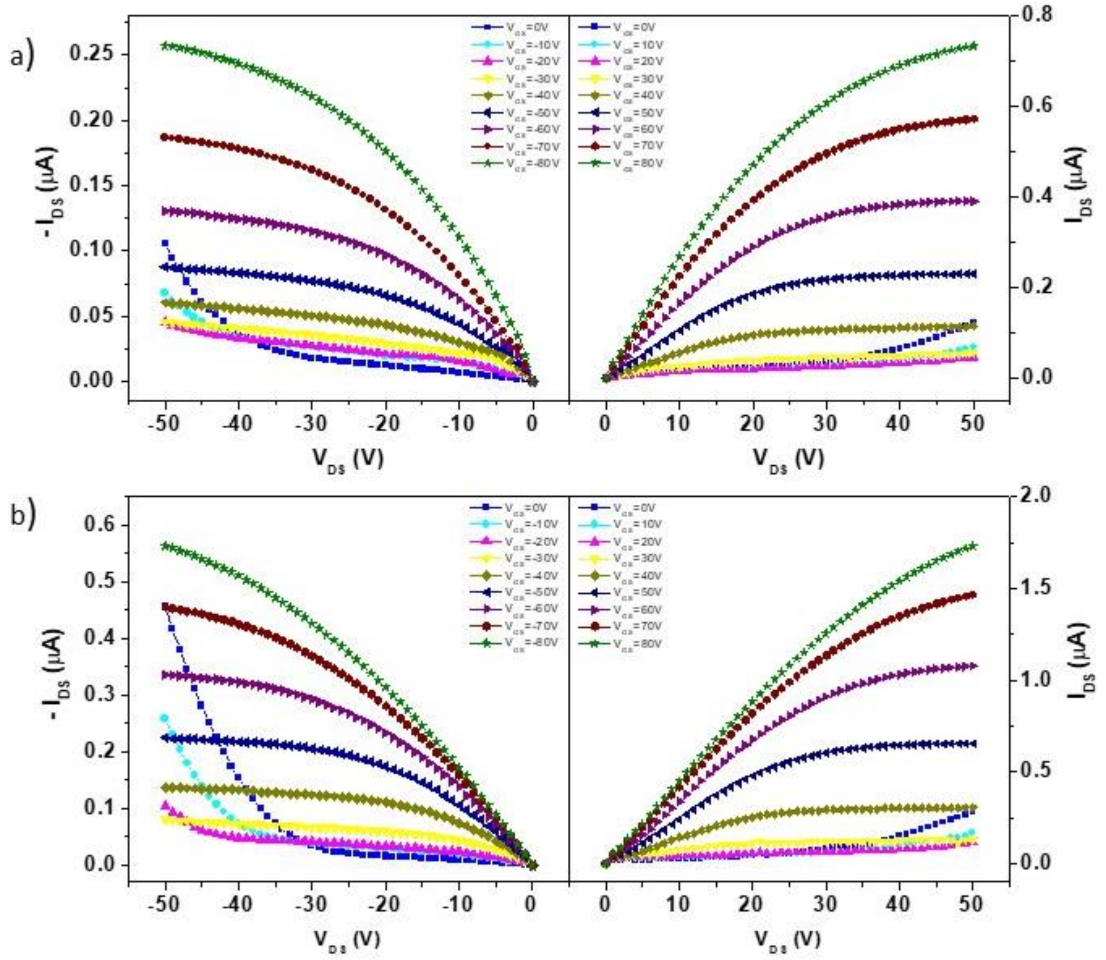

Figure S44. Output curves recorded for **A-DCV** (a) and **A-TB** (b) transistors under negative (on the left) or positive (on the right) V_{GS} and V_{DS} voltages.

Table S1: Thermal Properties of the A-series dyes

**Dyes**	T_m(°C)[a]	T_d(°C)[b]
**A-DCV**	155	348
**A-TB**	195	333
**A-ID**	212	366
**A-IDM**	227	340

a) Determined by DSC analysis run in N_2 atmosphere at 10 °C/min; b) determined by TGA analysis run in air at 10 °C/min; decomposition temperature is set as the temperature corresponding to 5 % weight loss.

Table S2. Optical properties in THF solution

**Dye**	**THF l_{max} (nm)**	**THF e ($dm^3 \cdot mol^{-1} \cdot cm^{-1}$)**
**A-DCV**	592/445	$4.51 \cdot 10^4/2.87 \cdot 10^4$
**A-TB**	616/473	$4.88 \cdot 10^4/3.12 \cdot 10^4$
**A-ID**	599/459	$4.46 \cdot 10^4/2.66 \cdot 10^4$
**A-IDM**	631/495	$4.85 \cdot 10^4/2.19 \cdot 10^4$

Table S3. Computed main optical transitions in the synthesized dye. Only transitions with Oscillator strength > 0.1 have been reported

Dye	Lambda_abs (nm)	Osc.strength
**A-DCV**	682.1688749	1.2716
	488.18440372	0.18020
	431.92542418	0.56550
	335.70030328	0.15980
**A-TB**	697.40236816	1.5668
	501.02720849	0.23110
	452.91029411	0.64280
**A-ID**	674.81735706	1.5968
	496.15508028	0.1574
	445.76182143	0.6102
**A-IDM**	744.33687346	1.4601
	546.30620406	0.2604
	498.18858445	0.3073
	470.88565519	0.2942
	361.36459636	0.1395
